# A mycovirus enhances fitness of an insect pathogenic fungus and potentially modulates virulence through interactions between viral and host proteins

**DOI:** 10.1371/journal.ppat.1013634

**Published:** 2025-10-23

**Authors:** Najie Shi, Guogen Yang, Ping Wang, Yulong Wang, Lili He, Rui Xie, Yang Yang, Deshui Yu, Robert H. A. Coutts, Ioly Kotta-Loizou, Bo Huang

**Affiliations:** 1 Anhui Provincial Key Laboratory of Microbial Pest Control, Anhui Agricultural University, Hefei, China; 2 Anhui Province Key Laboratory of Integrated Pest Management on Crops, School of Plant Protection, Anhui Agricultural University, Hefei, China; 3 Department of Clinical, Pharmaceutical and Biological Science, School of Health, Medicine and Life Sciences, University of Hertfordshire, College Lane Campus, Hatfield, United Kingdom; 4 Department of Life Sciences, Faculty of Natural Sciences, Imperial College London, South Kensington Campus, London, United Kingdom; Chinese Academy of Sciences, CHINA

## Abstract

*Beauveria bassiana* is an entomopathogenic ascomycete widely utilized in biological pest control. However, its effectiveness is often limited by low conidiation rates, sensitivity to environmental stresses, and delayed insecticidal activity. In this study, we identify and characterize a mycovirus, Beauveria bassiana polymycovirus 4–2 (BbPmV4-2), which markedly enhances the fitness and may modulate virulence of its fungal host. BbPmV4-2 comprises eight double-stranded RNA segments, among which three are unique and have not been previously detected in related mycoviruses. Infection with BbPmV4-2 nearly doubles conidial yields and upregulates key conidiation-related genes, facilitating enhanced dispersal of both the host fungus and the mycovirus itself. Additionally, BbPmV4-2 infected strains exhibit increased tolerance to ultraviolet (UV) irradiation and elevated temperatures, and may also exhibit increased virulence against the greater wax moth, *Galleria mellonella*. The potentially increased virulence is attributed to increased conidial hydrophobicity, adhesion, and cuticle penetration capabilities. Functional analysis reveals that the viral open reading frame ORF5 plays a critical role in conferring hypervirulence and stress tolerance by interacting with host proteins BbGAP1, a GPI-anchored membrane protein, and BbSDU1, a deubiquitinating enzyme. These interactions elucidate a molecular mechanism by which a mycovirus that enhances environmental adaptability and potentially influences host pathogenicity. Our findings provide significant insights into mycovirus-host interactions and suggest potential strategies for optimizing biological pest control applications.

## Author summary

Biological pest control using fungal pathogens offers a sustainable alternative to chemical pesticides, but current biocontrol agents often suffer from limitations including poor spore production, environmental sensitivity, and slow kill rates. Here, we report the discovery of a mycovirus—*Beauveria bassiana* polymycovirus 4–2 (BbPmV4-2)—that transforms its fungal host into a more effective biocontrol agent. This virus infects Beauveria bassiana, a widely used entomopathogenic fungus, and dramatically improves the fungus’s performance by nearly doubling spore production, enhancing survival under UV radiation and high temperatures, and accelerating insect killing. We demonstrate that these improvements stem from specific interactions between a viral protein (P5) and two key fungal proteins involved in membrane function and cellular regulation. Unlike many viruses that harm their hosts, BbPmV4-2 creates a mutually beneficial relationship. This discovery reveals how beneficial virus-host interactions can evolve and provides a potential strategy for developing improved biological pesticides. Understanding these mechanisms could lead to the engineering of enhanced fungal biocontrol agents that are more effective, resilient, and commercially viable, ultimately supporting more sustainable agricultural practices.

## Introduction

Mycoviruses, or fungal viruses, have been identified across a broad spectrum of fungal taxa since their initial discovery in the mushroom *Agaricus bisporus* [1]. Predominantly, these mycoviruses possess double-stranded (ds) RNA genomes and are currently classified into 17 families, including *Alternaviridae*, *Amalgaviridae*, *Botybirnaviridae*,*Chrysoviridae*, *Curvulaviridae*, *Fusagraviridae*, *Megabirnaviridae*, *Monocitiviridae*, *Megatotiviridae*, *Orthototiviridae*, *Phlegiviridae*, *Pseudototiviridae*, *Partitiviridae*, *Polymycoviridae*, *Quadriviridae*, *Spinareoviridae*, and *Yadonushiviridae* (https://ictv.global/taxonomy). Unlike many other viruses, mycoviruses typically do not have an extracellular phase in their replication cycle and are primarily transmitted vertically through asexual and sexual spores or horizontally via hyphal anastomosis [2,3].

The impact of mycovirus infection on fungal hosts is highly variable, ranging from deleterious effects to mutualistic interactions [4–7]. While many mycoviruses are associated with hypovirulence characterized by reduced mycelial growth, diminished conidiation, abnormal colony morphology, and disrupted cellular organization, others confer advantageous traits to their hosts [8–11]. For instance, the Saccharomyces cerevisiae virus L-A (ScVLA) induces the ‘killer yeast’ phenotype by supporting a satellite dsRNA that encodes a toxin, thereby providing a competitive advantage against sensitive strains [12]. Similarly, Pestalotiopsis theae chrysovirus 1 (PtCV1) transforms its pathogenic host into a non-pathogenic endophyte of tea leaves [13], and an *Alternaria alternata* mycovirus simultaneously represses fungal growth while enhancing virulence against pear plants [14]. These examples illustrate the complex interplay between mycoviruses and their fungal hosts, where viral infection can modulate host physiology and pathogenicity in diverse and context-dependent manners.

Entomopathogenic ascomycetes (EA) are important components of terrestrial microbiota, regulating arthropod populations in both epigeous and hypogeous habitats while establishing relationships with plants [15]. They inhabit soil environments and infected arthropod hosts as their primary ecological niches, providing multifaceted ecosystem services including direct and indirect pest biocontrol, promotion of plant growth, and enhancement of plant defenses [16–18].

The diversity of EA encompasses over 700 described species across multiple genera, with *Beauveria*, *Metarhizium*, *Lecanicillium*, and *Isaria* representing the most commercially developed lineages. These genera exhibit cosmopolitan distribution in natural and agricultural soils, with complex life cycles incorporating extended saprophytic phases during which conidia persist outside hosts [19,20]. Their ability to penetrate arthropod cuticles constitutes the fundamental mechanism underlying their biocontrol efficacy, making them valuable components of integrated pest management strategies [21,22].

Despite extensive taxonomic diversity, commercial development has focused on a narrow range of taxa, primarily *B. bassiana* and *M. anisopliae*. However, challenges remain in translating laboratory efficacy to field performance under variable environmental conditions [23]. Beyond direct pest control, EA provide ecosystem services including plant growth promotion and disease antagonism through their endophytic capacity, which induces changes in plant nutrient composition and defensive compounds [24,25].

*B. bassiana*, an entomopathogenic fungi, play a crucial role in biological pest control due to their broad-spectrum activity against various insect pests, including the pine caterpillar (*Thaumetopoea pityocampa*) and the corn borer (*Ostrinia furnacalis*) [26,27]. These fungi are integral to integrated pest management (IPM) strategies, effectively controlling pests like the coffee berry borer (*Hypothenemus hampei*) and the Colorado potato beetle (*Leptinotarsa decemlineata*) in agricultural settings [27,28]. Despite their efficacy, the commercial application of mycopesticides is often limited by factors such as low conidiation rates, sensitivity to environmental stresses, and slow insecticidal activity [29].

Mycoviruses have been identified in several entomopathogenic fungi, including *M. anisopliae* and *B. bassiana* [30–32]. In *M. anisopliae*, mycovirus infection typically results in decreased mycelial growth, reduced conidiation, lowered virulence, and impaired enzyme secretion [30,33]. Compared to mycoviruses in *Metarhizium* spp., viruses infecting *B. bassiana* have been studied more thoroughly, including investigations into their prevalence, identification, and the effects of mycoviruses on biological characteristics of this fungus [34–37]. Importantly, some mycoviruses in *B. bassiana* have been reported to enhance certain host traits. For example, infection with mycoviruses belonging to families *Chrysoviridae*, *Narnaviridae*, *Partitiviridae*, and *Polymycoviridae* has been associated with increased pigmentation, biomass, sporulation, stress tolerance, and virulence against the greater wax moth *G. mellonella* larvae [31,38–41]. These positive effects suggest that mycovirus-host interactions in entomopathogenic fungi may be harnessed to improve the efficacy of biological pest control agents [42,43].

Despite these promising observations, the molecular mechanisms by which mycoviruses enhance fungal fitness and virulence remain poorly understood [44]. Previous studies have shown that mycoviruses can modulate host gene expression by interacting with host proteins, influencing pathways related to stress response, sporulation, and pathogenicity [9,45–47]. For instance, the Cryphonectria parasitica hypovirus 1 (CHV1) papain-like protease p29 interacts with the host autophagy protein ATG8, suppressing sporulation and pigmentation [48]. Similarly, specific open reading frames (ORFs) in mycoviruses from *Magnaporthe oryzae* and *Aspergillus fumigatus* have been implicated in inhibiting conidial germination or modulating virulence-associated traits [47,49].

In this study, we characterize a mycovirus, BbPmV4-2, comprising eight dsRNA segments. Our findings demonstrate that BbPmV4-2 infection significantly enhances *B. bassiana* conidiation, tolerance to UV irradiation and heat shock, and could regulate virulence against *G. mellonella*. Through functional analyses of the viral ORFs, we identify P5 as a pivotal factor in conferring stress tolerance and potential hypervirulence by interacting with two host proteins: BbGAP1, a GPI-anchored membrane protein, and BbSDU1, a deubiquitinating enzyme. These interactions reveal a novel molecular mechanism by which a mycovirus may regulate environmental adaptability and host pathogenicity. Our study not only advances the understanding of mycovirus-host interactions but also highlights the potential of leveraging mycoviruses to enhance the efficacy of entomopathogenic fungi in biological pest control applications.

## Results

### Genome organization of BbPmV4-2

**Fig 1:**
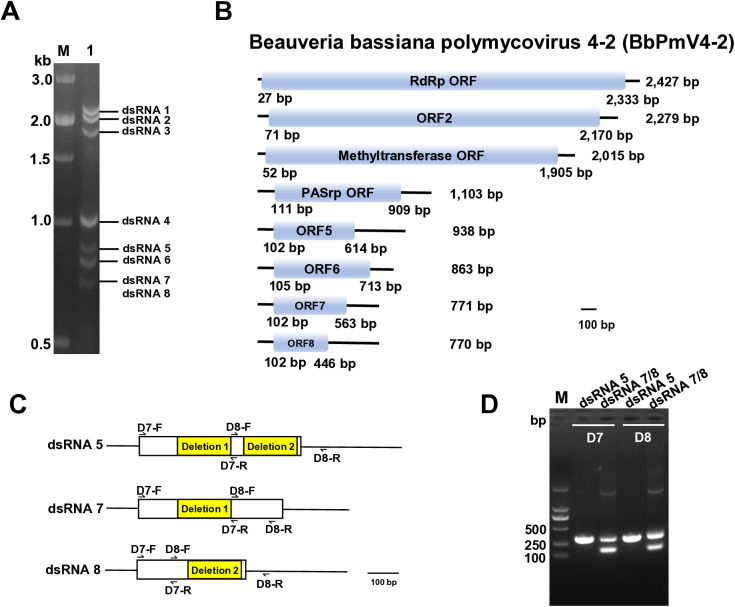
Genomic organization of BbPmV4-2. **(A)** Agarose gel electrophoresis of purified dsRNA extracted from *B. bassiana* strain Bb2151. Lane M, DNA molecular weight marker; Lane 1, dsRNAs of BbPmV4-2. **(B)** Schematic representation of the BbPmV4-2 genomic structure. ORFs (blue flanked by 5’ and 3’ untranslated regions (black lines)). **(C)** Schematic representation of the relationship between dsRNA 5 and the two defective dsRNAs 7 and 8. **(D)** Agarose gel electrophoresis of RT-PCR amplicons from purified dsRNA 5, dsRNA 7 and dsRNA 8, using sequence specific primers (D7-F/R and D8-F/R. Lane M, DNA molecular weight marker; Lane 1, RT-PCR using primers D7-F/R from dsRNA5; Lane 2, RT-PCR using primers D7-F/R from dsRNA 7 and dsRNA 8; Lane 3, RT-PCR using primers D8-F/R from dsRNA5; Lane 4, RT-PCR amplification using primers D8-F/R from dsRNA7 and dsRNA8). Primers used are listed in S1 Table.

Agarose gel electrophoresis of *B. bassiana* strain 2151 (Bb2151) extracts revealed the presence of eight distinct double-stranded RNA (dsRNA) segments, ranging in size from approximately 0.7 to 2.5 kbp (Fig 1A). Notably, the smallest dsRNA band comprises two nearly identical segments, designated as dsRNA 7 and dsRNA 8, each approximately 770 bp in length (Fig 1B). The full lengths of dsRNAs 1–8 are 2427, 2279, 2015, 1103, 938, 863, 771, and 770 bp, respectively. Each segment contains a single ORF on the positive-sense strand, which encodes proteins with estimated molecular masses of 83.14, 74.70, 66.43, 28.57, 18.80, 22.17, 17.30, and 12.14 kDa, respectively (Fig 1B). The sequences of all dsRNAs have been deposited in NCBI (GenBank accession numbers MZ328070-MZ328077).

Sequence analysis showed that all segments possess highly conserved 5′ and 3′ termini, supporting the notion that these eight dsRNAs together form the multipartite genome of a single virus, designated as BbPmV4-2 (S1A Fig). Comparative analysis showed that dsRNAs 1–4 and 6 of BbPmV4-2 share high nucleotide similarity with corresponding segments of BbPmV4, with similarities ranging from 98.56% to 99.46%. Specifically, dsRNAs 1–4 encode an RNA-dependent RNA polymerase (RdRp), a putative scaffold protein, a methyltransferase, and a proline-alanine-serine (PAS)-rich protein, respectively, while dsRNA 6 encodes a protein of unknown function (Fig 1B).

Distinctively, BbPmV4-2 possesses an additional segment, dsRNA 5, and two defective RNAs, dsRNA 7 and dsRNA 8. dsRNA 5 appears present in BbPmV4-2, but has not yet been identified in other BbPmV4 isolates. The amino acid sequence of dsRNA 5 exhibits 76.47% and 65.10% similarity to BbPmV3 and BbPmV2, respectively. Defective dsRNA 7 and dsRNA 8 originate from dsRNA 5, each resulting from deletions of 166 bp and 167 bp, respectively. This was confirmed by RT-PCR using specific primers D7-F/R and D8-F/R (Fig 1C and 1D). This is the first report of two defective RNAs identified within a polymycovirus.

Phylogenetic analysis using a maximum likelihood approach based on RdRp sequences places BbPmV4-2 firmly within the family *Polymycoviridae*, closely clustering with BbPmV4 (S1B Fig), underscoring its classification as a polymycovirus.

### BbPmV4-2 infection increases host fungal conidiation, improves tolerance to heat shock and UV irradiation, and potentially enhances virulence

To determine the phenotypic impact of BbPmV4-2 on *B. bassiana*, virus-free (VF) strains were derived from the original virus-infected (VI) strain Bb2151 via single-spore isolation. RT-PCR amplification targeting ORF1 and ORF5 confirmed the absence of BbPmV4-2 in thirty-two out of forty single-spore cultures (S2A-S2C Fig), from which three VF strains were randomly selected for subsequent analyses.

Equal volumes of spore suspensions (100 μL aliquots of a 1 × 10^7^ conidia/mL) of VF and VI were inoculated onto potato dextrose agar (PDA) plates, and incubated for 7 d, 10 d or 14 d to quantify conidial production. Conidiation assays revealed that VI strains produced nearly twice as many conidia as their VF counterparts (S3A and S3B Fig). RT-qPCR analysis showed a two-fold upregulation of the *fluG* gene and significant upregulation of all *flb* genes, with *flbE* exhibiting a three-fold increase in expression in VI strains compared to VF strains (S3C Fig). These findings indicate that BbPmV4-2 infection substantially enhances fungal conidiation, thereby promoting the dispersal of both the host fungus and the mycovirus itself.

Conversely, conidia (1 µL from 1 × 10^6^ conidia/mL suspension) were inoculated at the centre of sabouraud dextrose agar with yeast extract (SDAY) plates, and colony diameter was measured after 10 d of incubation to assess radial growth. No significant differences were observed in radial growth between VI and VF strains on SDAY, 1/4 SDAY, and PDA plates (S3D and S3E Fig). Both VI and VF strains exhibited similar responses to high salinity, Congo red (which disrupts cell wall integrity), and oxidative stress (S3F and S3G Fig). These results suggest that BbPmV4-2 does not influence host tolerance to these specific chemical stresses.

**Fig 2:**
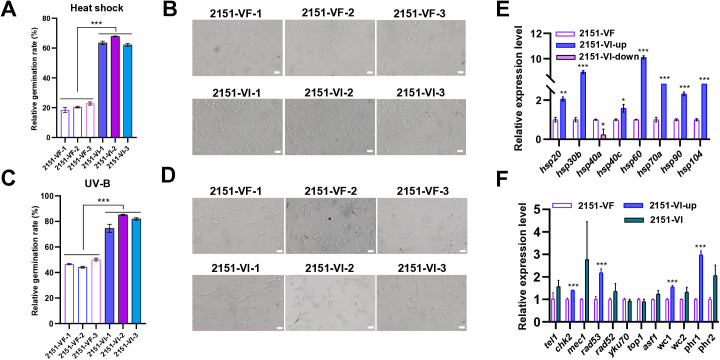
Impacts of BbPmV4-2 on heat and UV stress responses. **(A)** Relative conidial germination rate and (B) microscopic images (scale bar 20 μm) of VF and VI strains on PDA plates 24 h after exposure to 42 °C for 60 min. **(C)** Relative conidial germination rate and (D) microscopy images (scale bar: 20 μm) of VF and VI strains on PDA plates 24 h after exposure to 100 mJ/cm^2^ UV for 10 sec. Relative transcript levels in VF and VI strains of (E) eight hsp genes and (F) twelve DNA damage repair genes, as measured by RT-qPCR. For each gene, expression in VF was set as 1 and relative expression in VI was calculated. Primers used are listed in S2 Table. Data were present as the mean ± standard deviation (SD) from three replications. Student’s t-test or ANOVA, * *P* < 0.05, ** *P* < 0.01 or *** *P* < 0.001.

The resilience of BbPmV4-2-infected strains to environmental stressors was further evaluated by assessing conidial germination rates following exposure to heat shock and UV irradiation. Conidia were inoculated onto PDA plates, and the germination rate was evaluated every 4 h up to 24 h by counting the number of germinated conidia under a microscope. Under 42 °C heat shock conditions, VI strains exhibited a significant increase in relative germination rate, reaching 65% as compared to 21% in VF strains (Fig 2A and 2B). Similarly, after exposure to 100 mJ/cm^2^ UV for 10 sec, VI strains showed a relative germination rate of 81%, markedly higher than the 47% observed in VF strains (Fig 2C and 2D).

RT-qPCR revealed that VI strains upregulated a suite of heat shock protein (HSP) genes and DNA repair genes, including *hsp20*, *hsp30b*, *hsp40c*, *hsp60*, *hsp70a*, *hsp90*, *hsp104*, *chk2*, *rad53*, *wc1*, and *phr1* (Fig 2E and 2F). The upregulation of these genes, particularly the several-fold increase of *hsp60* and DNA repair genes such as *rad53* and *phr1*, underpins the enhanced tolerance to heat and UV stress observed in VI strains. These molecular changes suggest that BbPmV4-2 infection augments the host fungus’s ability to withstand and repair damage from environmental stressors, thereby enhancing its adaptability and survival in adverse conditions.

**Fig 3:**
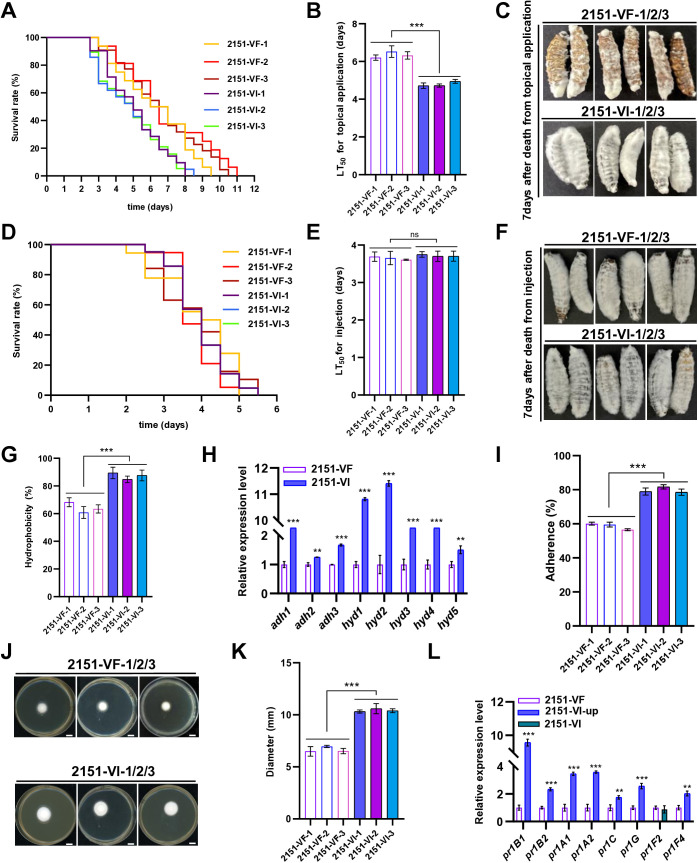
Impacts of BbPmV4-2 on virulence. **(A)** Survival of *G. mellonella* larvae, **(B)** LT_50_ and (C) mycosis after cuticular infection with VF and VI strains. **(D)** Survival of *G. mellonella* larvae, **(E)** LT_50_ and (F) mycosis after injection with VF and VI strains. Control group (CK) larvae were treated with sterile water. **(G)** Conidial hydrophobicity rates of VF and VI strains. **(H)** Relative expression levels in VF and VI strains of five hydrophobicity genes and three conidial adherence genes, as measured by RT-qPCR. **(I)** Conidial adherence of VF and VI strains. **(J)** Growth of VF and VI strains on SDAY plates following penetration of cicada wings (scale bar 6 mm) and (K) quantification of the conidial penetration ability of VF and VI strains. **(L)** Relative expression levels in VF and VI strains of eight cuticle degradation and virulence genes, as measured by RT-qPCR. For each gene, expression in VF was set as 1 and relative expression in VI was calculated. Data were present as the mean ± standard deviation (SD) from three replications. Student’s t-test or ANOVA, * *P* < 0.05, ** *P* < 0.01 or *** *P* < 0.001.

To assess the impact of BbPmV4-2 on fungal virulence, *G. mellonella* larvae were used as a model host. In cuticular infection assays, VI strains significantly reduced the median lethal time (LT_50_) to 4.8 ± 0.13 d as compared to 6.4 ± 0.16 ds for VF strains (Fig 3A and 3B). Additionally, larvae infected with VI strains exhibited extensive mycosis and sporulation on their cadavers, characterized by a thick layer of conidia (Fig 3C).

Conversely, no significant differences in LT_50_ were observed between VI and VF strains following intrahaemocoelic injection (Fig 3D-3F). This suggests that BbPmV4-2 may enhance virulence primarily by facilitating the early stages of cuticular infection rather than systemic infection following penetration.

To elucidate the mechanisms underlying the observed hypervirulence, we examined the biophysical properties of conidia. VI conidia exhibited significantly higher hydrophobicity (87%) compared to VF conidia (64%) (Fig 3G). Correspondingly, transcript levels of five hydrophobin-related genes (*hyd1*-*hyd5*) were upregulated up to ten-fold in VI strains, suggesting enhanced hydrophobin synthesis contributes to increased hydrophobicity (Fig 3H).

Adhesion assays revealed that VI conidia had an adhesion rate of 80%, significantly higher than the 59% observed in VF conidia (Fig 3H and 3I). Additionally, the expression of three adhesion-related genes (*adh1*, *adh2*, and *adh3*) was upregulated up to five-fold in VI strains. Notably, *adh2* is known to facilitate conidial adhesion to the insect cuticle in association with adhesion-related genes.

Penetration assays demonstrated that VI conidia possessed a significantly higher penetration ability compared to VF conidia (Fig 3J and 3K). This enhanced penetration correlates with the upregulation of seven out of eight cuticle-degrading and virulence-associated genes, including those encoding multiple proteases (*pr1A1*, *pr1A2*, *pr1C*, *pr1G*, and *pr1F4*) and cuticle-degrading proteinases (*pr1B1* and *pr1B2*) (Fig 3L). Among these, *pr1B1* and *pr1B2* are established virulence factors, with *pr1B1* showing an eight-fold upregulation in VI strains.

Collectively, these data indicate that BbPmV4-2 infection enhances conidial hydrophobicity, adhesion, and penetration, thereby promoting efficient cuticular infection and contributing to the hypervirulent phenotype observed in VI strains.

To dissect the contributions of individual viral proteins to the enhanced phenotypes, we generated *B. bassiana* strains expressing each of the eight BbPmV4-2 ORFs (*ex-ORF1* to *ex*-*ORF8*) and confirmed their expression via PCR, RT-PCR, RT-qPCR, and immunoblotting (S4A-S4D Fig). All ORFs, except ORF6, were successfully expressed at the protein level.

**Fig 4:**
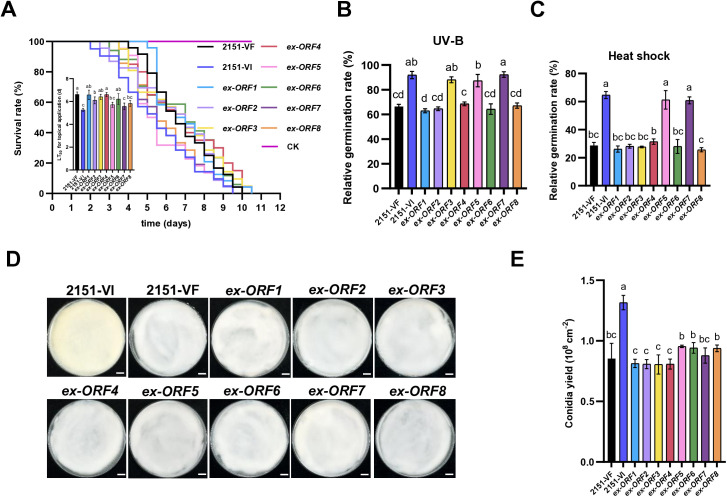
Impact of BbPmV4-2 ORFs 1-8 on *B. bassiana.* **(A)** Survival of *G. mellonella* larvae and LT_50_ after cuticular infection with VF, VI, and *ex-ORF*s 1-8 strains. **(B)** Relative conidial germination rate of VF, VI and *ex-ORF*s 1-8 strains on PDA plates 24 h after exposure to 100 mJ/cm^2^ UV for 10 sec. **(C)** Relative conidial germination rate of VF, VI and *ex-ORF*s 1-8 strains on PDA plates 24 h after exposure to 42 °C for 60 min. **(D)** Colony morphology of VF, VI and *ex-ORF*s 1-8 strains (25 °C, 10 dpi). Scale bar = 6 mm. **(E)** Conidial yields of VF, VI and *ex-ORF*s 1-8 strains 10 dpi. Data were present as the mean ± standard deviation (SD) from three replications. Student’s t-test or ANOVA, Groups with no significant differences share the same letter, while those with significant differences are labeled with different letters.

Phenotypic analyses of these ORF-expressing strains revealed that *ex-ORF5*, *ex-ORF7*, and *ex-ORF8* significantly reduced the LT_50_ in *G. mellonella* larvae compared to the VF strain, with LT_50_ values of 5.7 ± 0.26, 5.6 ± 0.29, and 5.7 ± 0.06 d, respectively (Fig 4A). Both dsRNA7 and dsRNA8 are variants of dsRNA5, emphasizing that ORF5 likely plays a key role in increasing virulence.

Further assessments demonstrated that *ex-*ORF*5* strains exhibited a 32% and 114% increase in conidial relative germination rates following UV irradiation and heat shock, respectively (Fig 4B and 4C). Similarly, ORF7 expression conferred a 39% and 113% increase in relative germination rates under UV and heat stress. Additionally, ORF3 was identified as a specific enhancer of UV resistance, with a 33% increase in relative germination rates post-UV exposure (Fig 4B). However, ORF3 did not impact insecticidal efficacy, sporulation, or heat shock resistance, indicating that multiple viral proteins contribute to the overall stress tolerance phenotype (Fig 4A, 4C, and 4E).

### The BbPmV4-2 P5 protein interacts with host proteins such as BbGAP1, which is membrane-associated, and BbSDU1, which is vacuole-associated

**Fig 5:**
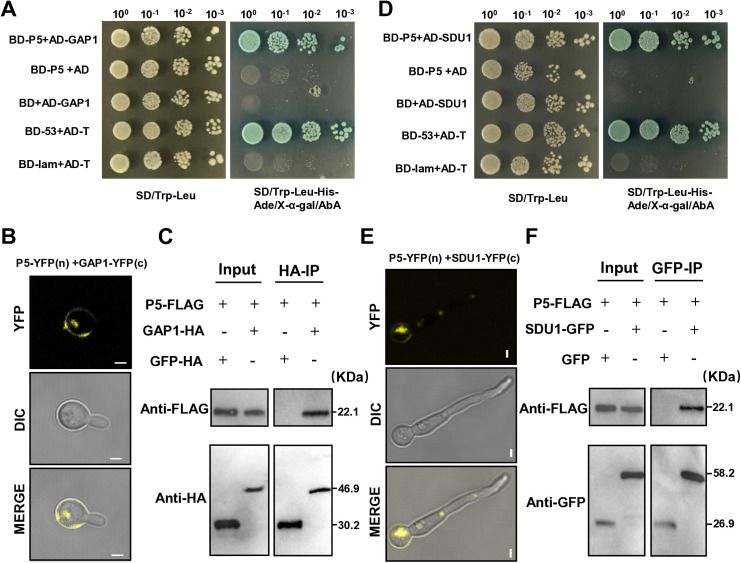
Interaction between P5 and BbGAP1, and between P5 and BbSDU1 in *B. bassiana.* **(A)** Y2H analysis of the interaction between P5 and BbGAP1. **(B)** BiFC assays for detecting in vivo protein interactions (scale bar 1 μm). **(C)** Co-IP assay of P5 with BbGAP1. IP(HA), immunoprecipitation with FLAG and HA. **(D)** Y2H analysis of the interaction between P5 and BbSDU1. **(E)** BiFC assays for detecting in vivo protein interactions (scale bar 1 μm). **(F)** Co-IP assay of P5 with BbSDU1. IP(GFP), immunoprecipitation with FLAG and GFP. Primers used are listed in S4 Table.

To identify host factors interacting with the viral protein P5, yeast two-hybrid (Y2H) screening of a *B. bassiana* cDNA library identified 18 potential interacting proteins (S3 Table). Subsequent validation using Yeast two-hybrid (Y2H) assays, bimolecular fluorescence complementation (BiFC), and co-immunoprecipitation (Co-IP) confirmed that P5 physically interacts with two host proteins: BbGAP1, a GPI-anchored membrane protein (XP_008599458), and BbSDU1, a deubiquitinating enzyme (XP_008601556) (Fig 5A-5F).

The BbGAP1 protein, identified through Y2H screening, was further characterized. The cloned gene segment is 1,296 bp, contains a single exon, and encodes a 431 amino acid protein with a predicted molecular mass of 43.48 kDa and an isoelectric point of 5.72. Sequence analysis revealed significant homology (65.54-72.83% similarity) to known fungal GAP1 proteins from various species. BbGAP1 contains an N-terminal signal peptide and a C-terminal GPI-attachment signal region, along with a predicted transmembrane domain. Phylogenetic analysis positions BbGAP1 within a clade of fungal GAP1 proteins, supporting its classification as the characterized *Gap1* gene in *B. bassiana* (S5A and S5B Fig).

A construct combining BbGAP1 with a GFP gene fluoresced brightly in both vegetative hyphae and spores (S5C Fig), further suggesting that BbGAP1 is a cell membrane associated protein as predicted by sequence analysis.

**Fig 6:**
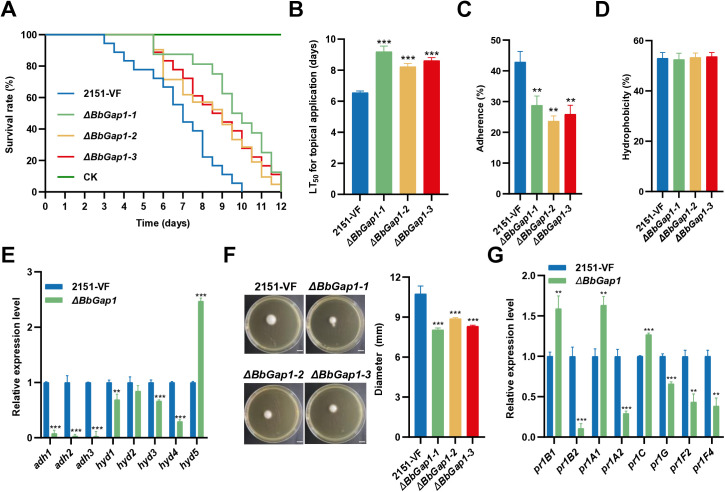
Impacts of *BbGap1* on virulence and conidial adhesion, hydrophobicity, and penetration. **(A)** Survival of larvae and **(B)** LT_50_ after cuticular infection with VF and *ΔBbGap1* strains. CK larvae were treated with sterile water. **(C)** Conidial adherence of VF and *ΔBbGap1* strains. **(D)** Conidial hydrophobicity rates of VF and *ΔBbGap1* strains. **(E)** Relative expression levels in VF and *ΔBbGap1* strains of five hydrophobicity genes and three conidial adherence genes, as measured by RT-qPCR. **(F)** Growth of VF and *ΔBbGap1* strains on SDAY plates following penetration of cicada wings (scale bar 6 mm) and quantification of the conidial penetration ability of VF and *ΔBbGap1* strains. **(G)** Relative expression levels in VF and *ΔBbGap1* strains of eight cuticle degradation and virulence genes, as measured by RT-qPCR. For each gene, expression in VF was set as 1 and relative expression in VI was calculated. Data were present as the mean ± standard deviation (SD) from three replications. Student’s t-test or ANOVA, * *P* < 0.05, ** *P* < 0.01 or *** *P* < 0.001.

Deletion of *BbGap1* in *B. bassiana* (S6A-S6E Fig) resulted in significantly reduced virulence in cuticular infection assays, with LT_50_ values increasing to 8.2-9.2 d compared to 6.6 d in the VF strain (Fig 6A and 6B). However, significant difference was observed in LT_50_ following intrahaemocoelic injection (S7A and S7B Fig). While hydrophobicity was unaffected, conidial adhesion to locust wing cuticles was significantly reduced by 3345% in the mutants (Fig 6C and 6D), accompanied by downregulation of the adhesion-related *adh1*, *adh2*, *adh3* genes and alterations in hydrophobin gene expression (Fig 6E). *BbGap1* deletion mutants also showed decreased conidial penetration ability (Fig 6F) and downregulation of the *pr1B2*, *pr1A2*, *pr1G*, *pr1F2*, and *pr1F4* genes (Fig 6G). Based on the current findings, it can be hypothesized that P5 may enhance virulence through interaction with BbGAP1 and influencing its downstream pathways.

Similarly, BbSDU1 was identified as an interacting partner of P5 through Y2H screening. The *BbSdu1* gene is 945 bp, containing three exons and two introns, encoding a 284 amino acid protein with a molecular mass of 31.25 kDa and an isoelectric point of 9.0. Phylogenetic analysis clusters BbSDU1 with deubiquitinating enzymes from related entomopathogenic fungi. Subcellular localization assays indicated that BbSDU1 localizes to the vacuole (S8A-S8C Fig).

**Fig 7:**
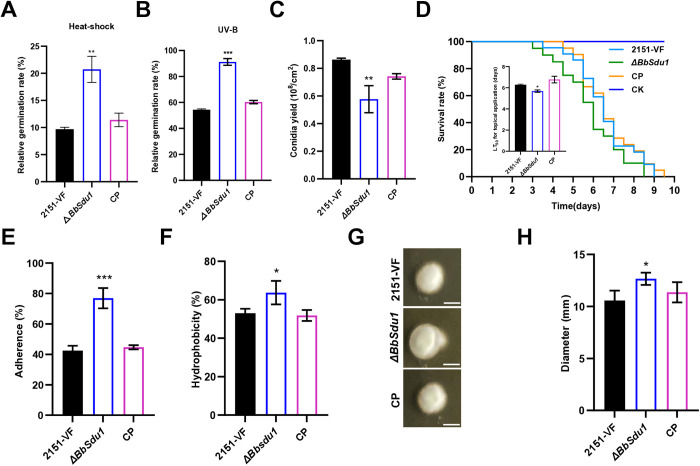
Impacts of *BbSdu1* on virulence and fitness of *B.bassiana.* **(A)** Relative conidial germination rate of VF and *ΔBbSdu1* strains on PDA plates 24 h after exposure to 42 °C for 60 min. **(B)** Relative conidial germination rate of VF and *ΔBbSdu1* strains on PDA plates 24 h after exposure to 100 mJ/cm^2^ UV for 10 sec. **(C)** Conidial yields of VF and *ΔBbSdu1* strains 10 dpi. **(D)** Survival of larvae and LT_50_ after cuticular infection with VF and *ΔBbSdu1* strains. CK larvae were treated with sterile water. **(E)** Conidial adherence of VF and *ΔBbSdu1* strains. **(F)** Conidial hydrophobicity rates of VF and *ΔBbSdu1* strains. **(G)** Growth of VF and *ΔBbSdu1* strains on SDAY plates following penetration of cicada wings (scale bar 6 mm) and (H) quantification of the conidial penetration ability of VF and *ΔBbSdu1* strains. Data were present as the mean ± standard deviation (SD) from three replications. Student’s t-test or ANOVA, * *P* < 0.05, ** *P* < 0.01 or *** *P* < 0.001.

Deletion of *BbSdu1* (S9A to S9F Fig) resulted in increased tolerance to heat shock and UV-B irradiation, as evidenced by higher conidial germination rates in *ΔBbSdu1* strains compared to VF and complemented (CP) strains (Fig 7A and 7B). Additionally, *ΔBbSdu1* strains exhibited reduced sporulation (Fig 7C). In virulence assays, *ΔBbSdu1* strains showed significantly increased virulence in cuticular infection, with an LT_50_ of 5.7 ± 0.11 d compared to 6.3 ± 0.06 and 6.8 ± 0.28 d for VF and CP strains, respectively (Fig 7D). However, no significant changes were observed in virulence following intrahaemocoelic injection (S10A and S10B Fig).

*ΔBbSdu1* strains also displayed increased hydrophobicity (64% vs. VF and CP) and enhanced conidial adhesion (77% vs. 42.5-44.7% in VF and CP) (Figs 7E, 7F and S10 C). Penetration assays showed successful colony formation on cicada wings (Fig 7G and 7H), and upregulation of *pr1B1*, *pr1A2*, *pr1C*, and *pr1F2* genes was observed (S10D Fig). These findings suggest that BbSDU1 acts as a negative regulator of stress response and potential virulence-related traits, and its interaction with viral protein P5 may modulate these functions to enhance fungal fitness.

Furthermore, ubiquitination assays revealed elevated ubiquitination levels of ORF5 in the ORF5-overexpressing strain (VF-ORF5^oe^), indicating that ORF5 may suppress the deubiquitinating activity of BbSDU1, thereby altering the ubiquitination status of host proteins and contributing to enhanced fungal fitness (S11A Fig).

## Discussion

In this study, we have identified and characterized a polymycovirus (BbPmV4-2), which comprises eight dsRNA segments. BbPmV4-2 is among the few members of the *Polymycoviridae* family exhibiting an eight-segmented genome, akin to Colletotrichum camelliae filamentous virus 1 (CcFV1) [50,51] and Fusarium redolens polymycovirus 1 (FrPmV1) [52]. Notably, BbPmV4-2 possesses an additional dsRNA segment (dsRNA 5) and two defective RNAs (dsRNA 7 and dsRNA 8) not present in its close relative BbPmV4. This is the report documenting defective RNAs within a polymycovirus, suggesting potential mechanisms of genome evolution such as horizontal gene transfer or segment loss. The presence of filamentous virions in CcFV1 raises intriguing questions about the relationship between virion morphology and genome complexity, although the virion structures of FrPmV1 and BbPmV4-2 remain to be elucidated [51,52].

Functionally, BbPmV4-2 infection markedly enhances several key phenotypic traits of its host, *B. bassiana*. The nearly twofold increase in conidiation observed in virus-infected (VI) strains aligns with previous reports where polymycovirus infections led to enhanced sporulation in other fungal species [38]. This enhancement is underpinned by the upregulation of central developmental pathway genes *brlA* and *abaA* [53,54], which are critical for asexual reproduction [55]. Enhanced conidiation not only facilitates greater dispersal of the fungus but also ensures efficient transmission of the mycovirus [4,56,57], thereby establishing a mutualistic relationship that benefits both organisms [5].

Crucially, BbPmV4-2 confers increased tolerance to environmental stressors, specifically heat shock and UV irradiation. RT-qPCR analyses revealed significant upregulation of *hsp* genes and DNA repair enzymes in VI strains, providing a molecular basis for the observed phenotypic resilience. This enhanced stress tolerance is particularly advantageous for *B. bassiana* in field conditions, where fluctuating temperatures and UV exposure can limit fungal efficacy as a biocontrol agent [58,59].

The effectiveness of fungal biopesticides is largely dependent on the spores of entomopathogenic fungi, which act through a multifaceted mechanism against target pests. The pathogenic process consists of two main stages: (1) attachment and penetration of the insect cuticle, and (2) colonization of the hemocoel and systemic infection. Initially, fungal spores attach to the surface of the insect cuticle and then initiate enzymatic reactions that enable them to breach the chitinous exoskeleton. After entering into the host, the fungus undergoes rapid proliferation and releases bioactive toxins, ultimately resulting in the death of the insect. The process of penetrating the insect cuticle typically requires a longer duration than direct hemocoel injection [60]. Perhaps most notably, BbPmV4-2 induces potential hypervirulence against *G. mellonella* larvae during cuticular infection. This is achieved through multiple mechanisms: increased conidial hydrophobicity, enhanced adhesion to the insect cuticle, and improved penetration capabilities [60–62]. The upregulation of hydrophobin and adhesion-related genes, along with cuticle-degrading proteases, collectively contribute to the accelerated pathogenicity observed in VI strains. Importantly, our functional analyses pinpoint ORF5 as a critical determinant of stress tolerance and of possible hypervirulence. P5 interacts directly with two host proteins, BbGAP1 and BbSDU1, potentially modulating host cellular processes to favor enhanced fungal fitness and pathogenicity.

The interaction between P5 and BbGAP1, a GPI-anchored membrane protein, underscores a novel mechanism by which mycoviruses can influence host cell surface properties and adhesion dynamics [63]. Conversely, the interaction with BbSDU1, a deubiquitinating enzyme, suggests a role for viral proteins in modulating the host’s protein ubiquitination status, thereby affecting stress response pathways and virulence factors [64]. These interactions not only elucidate the molecular underpinnings of mycovirus-induced hypervirulence but also reveal potential targets for genetic or chemical manipulation to further enhance fungal biocontrol efficacy. Furthermore, molecular docking analysis with ZDOCK (http://zdock.umassmed.edu/) indicated that GAP1 is capable of interacting with P5, P7, and P8, but each interaction occurs at different binding sites. However, SDU1 only interacts with P5, with no detectable interaction sites for P7 or P8 [65]. These results indicate that although P5, P7, and P8 share partial amino acid sequence overlap, their protein structures and functional domains differ. Consequently, in *B. bassiana*, fungal proteins interacting with P5 likely exhibit divergent interaction mechanisms compared to those involving P7 and P8.

Our findings extend the current understanding of mycovirus-host interactions by demonstrating that viral proteins can directly manipulate host cellular machinery to confer advantageous traits. This contrasts with the more commonly observed hypervirulent‌ effects of mycovirus infections and highlights the potential of harnessing beneficial mycoviruses to improve the performance of entomopathogenic fungi in agricultural settings. Moreover, the discovery of defective RNAs within BbPmV4-2 opens new avenues for exploring viral genome plasticity and its impact on host interactions.

While our study confirms the influence of the mycovirus on fungal fitness and suggests its potential involvement in pathogenicity, we acknowledge certain limitations in our current approach, particularly the lack of a comprehensive log-dose-probit mortality analysis across different concentration gradients. Future investigations could utilize multi-dose bioassays along with appropriate statistical modeling to achieve more definitive conclusions about the effect of the mycovirus on fungal virulence.

## Conclusion

**Fig 8:**
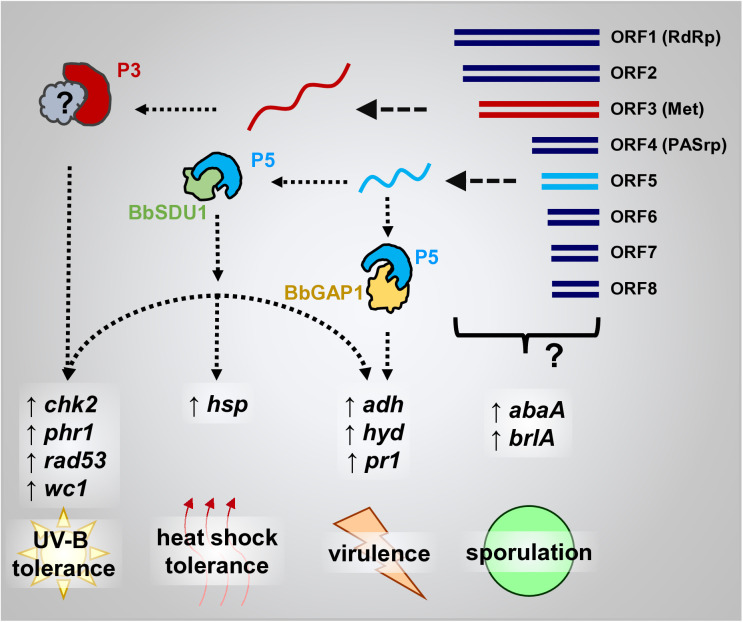
Mechanistic model illustrating how mycovirus infection enhances the fitness of *B. bassiana.* Each BbPmV4-2 genomic segment expresses viral proteins. The viral protein P3 interacts with *B. bassiana* proteins, upregulating genes involved in UV-B response and enhancing UV-B tolerance. The viral protein P5 interacts with *B. bassiana* proteins (BbGAP1 and BbSDU1), upregulating genes involved in UV-B and heat shock response, and virulence. This interaction enhances UV-B and heat shock tolerance, and increases the insecticidal ability. Although individual viral proteins were not shown to affect sporulation, BbPmV-4-2 infection as a whole contributes to increased sporulation.

We have identified and elucidated the role of BbPmV4-2, an eight-segmented mycovirus, in enhancing the fitness and potential virulence of its entomopathogenic host, *B. bassiana*. BbPmV4-2 infection significantly augments key fungal traits, including conidiation, tolerance to heat shock and UV irradiation, and may modulate virulence against *G. mellonella* larvae. These enhancements are mediated through the upregulation of stress-responsive and virulence-associated genes, as well as through direct interactions between the viral protein P5 and host proteins BbGAP1 and BbSDU1 (Fig 8).

The discovery of BbPmV4-2’s unique genomic structure, including its defective RNAs, and its ability to confer hypervirulence represents a significant advancement in our understanding of mycovirus biology and their potential applications. The elucidated molecular mechanisms provide a foundation for future studies aimed at optimizing mycovirus-mediated enhancements for biological pest control. By leveraging such mutualistic virus-host interactions, it is possible to develop more effective and resilient biocontrol agents, thereby offering sustainable solutions to manage agroforestry arthropod pests.

These findings not only broaden the scope of mycovirus research in entomopathogenic fungi but also pave the way for innovative strategies to harness viral symbionts in agricultural biotechnology. Ultimately, BbPmV4-2 exemplifies the potential of beneficial mycoviruses to transform the efficacy of fungal biocontrol agents, contributing to more robust and adaptable pest management systems.

## Materials and methods

### Fungal strains and culture conditions

*B. bassiana* strain 2151 (Bb2151) was isolated from a treehopper of the family *Membracidae* in Anhui province, China, and it together with all other fungal strains were cultured on sabouraud dextrose agar with yeast extract (SDAY) (1% w/v peptone, 0.2% w/v yeast extract, 4% w/v dextrose, and 1.5% w/v agar) at 25 °C with a 12 h light/12 h dark photoperiod for up to 12 d unless specified otherwise [66].

BbPmV4-2 was cured from Bb2151 using the single-spore isolation method, as described previously [67]. The resultant cultures were verified for the presence or absence of the virus following of RNA extraction and RT-PCR, resulting in the generation of respectively VI and VF single-spore isogenic lines.

### RNA extraction, reverse transcription (RT) and quantitative polymerase chain reaction (qPCR)

Total RNA was extracted with the TRIzol reagent (Invitrogen). BbPmV4-2 dsRNA was extracted and purified using CF-11 cellulose (Sigma) chromatography, as described previously [68], and the dsRNA obtained was purified further following DNase I and S1 nuclease digestion. For next-generation sequencing, 5 µg of dsRNA was used to construct cDNA libraries for the Illumina HiSeq 2500 platform at BGI (Shenzhen, China). The 5′ and 3′ ends of dsRNAs were determined using RNA-ligase-mediated rapid amplification of cDNA ends (RLM-RACE) [69]. To verify the presence or absence of BbPmV4-2, RT of total RNA or dsRNA was performed using Moloney Murine Leukemia Virus (M-MLV) transcriptase (TaKaRa, Dalian, China), followed by PCR with sequence specific primers (S1 Table). For RT-PCR, complementary DNA (cDNA) was synthesized using the ReverTra Ace qPCR RT master mix with gDNA remover kit (Toyobo, Japan), according to the manufacturer’s instructions. Real-time qPCR was performed using the qPCR SYBR green master mix (Vazyme, China) and sequence specific primers (S2 Table) in the Bio-Rad CFX96 real-time PCR detection system (Bio-Rad, USA). The relative expression levels of genes were determined via the 2^-ΔΔ*Ct*^ method with *β-actin* (BBA_04860) as the reference gene [70].

### Bioinformatics analysis

Reads generated by next-generation sequencing were processed prior to assembly and analysis according to protocols described previously [71]. Multiple alignment based on the RdRp amino acid sequence of BbPmV4-2 and other mycoviruses was performed using the Multiple Alignment Fast Fourier Transform (MAFFT) program [72], and the phylogenetic tree was constructed by the MEGA X program using the maximum-likelihood (ML) method with the LG + G + F model and 1000 bootstrap replicates [73].

### Plasmid construction and fungal transformation

Full-length coding regions of ORFs 1–8 were cloned into the pDHt-SK-Bar-PgpdA vector to obtain pDHt-SK-Bar-PgpdA-ORF 1/2/3/4/5/6/7/8-FLAG, respectively. The ORF expressing mutants were generated by Agrobacterium tumefaciens mediated transformation (ATMT) and confirmed by PCR, RT-PCR, RT-qPCR, and immunoblotting [74,75]. All primers used are listed in S5 and S6 Tables.

Gene replacement of *BbGap1/BbSdu1* was conducted by homologous recombination of pDHt-SK-Bar vector in the wild type strain using ATMT. Putative mutant colonies were screened for resistance to phosphinothricin (200 mg/mL). All primers used are listed in S7 Table.

The target sequence from the 5’-terminal region of ORF5 was chosen for RNA interference. The RNAi vector was designed with the following arrangement: pDHt-SK-Bar-PgpdA- [target sequence]-loop sequence- [reverse-complementary target sequence]-Ttrpc. This construct was introduced into the VI strain using ATMT, and putative mutants were selected based on resistance to phosphinothricin (200 mg/mL). All primers used are listed in S7 Table.

### Y2H, BiFC and Co-IP assays

For library construction, RNA was isolated from Bb2151 cultured in MM (0.1%KH_2_PO_4_, 0.1%MgSO_4_.7H_2_O and 50% tap-water) with locust body walls for 8 h [76]. The Y2H library was constructed and cloned into the pGADT7 vector by OE Biotech (Shanghai, China). For library screening, the ORF5 bait construct was cloned into the pGBKT7 vector. Yeast colonies that grew on SD-Trp-Leu-His-Ade and had β-galactosidase activity were isolated as putative P5-interacting proteins (OIPs). To directly confirm such interactions, full-length coding regions of OIP fragments were amplified and cloned into pGADT7 for further Y2H assays [77]. All primers used are listed in S4 Table.

The cDNA of ORF5 and OIPs were introduced into YFPC or YFPN to generate the P5-YFPN and OIPs-YFPC plasmids. The plasmids were subsequently transformed into Bb2151 through ATMT. The strains were confirmed by PCR [78]. All primers used are listed in S4 Table. The mutants were observed under a confocal microscope (Zeiss LSM 980, Lena, Germany).

The P5-FLAG and OIPs-HA/GFP were co-transformed into Bb2151. Total protein was extracted from the transformed strains and incubated with Anti-HA/GFP Tag mAb Agarose conjugated (Abmart, Shanghai, China) at 4 °C for 3 h. Subsequently, tagged proteins was detected by immunoblotting using HA (Abmart, Shanghai, China)/GFP (Abmart, Shanghai, China) and FLAG (Abmart, Shanghai, China) antibodies, respectively [79]. All primers used are listed in S4 Table.

### Subcellular localization assay

To determine its subcellular localization, full-length coding regions of BbGAP1/BbSDU1 was fused with GFP at its C terminus. The fusion gene cassettes were cloned into the pDHt-Ben vector under the control of the gpdA promoter [80]. All primers used are listed in S4 Table.

### Assays for fungal growth, conidiation and conidial germination

For growth assays, conidia (1 μL aliquots from 1 × 10^6^ conidia/mL suspensions) were inoculated at the centre of SDAY, 1/4 SDAY, and PDA plates, and radial growth (colony diameter) was measured after 10 d incubation. For conidiation assays, 100 μL aliquots of a 1 × 10^7^ conidia/mL suspension were spread on PDA plates and incubated for 7 d, 10 d, or 14 d to assess conidia production. Fresh conidia from each culture were dispersed in 30 mL of 0.05% (v/v) Tween 80 following vortexing, and the concentration of conidia of the suspension determined using a hemocytometer. For conidial germination assays, conidia (20 μL from 5 × 10^5^ conidia/mL suspensions) were spread on PDA plates. The percentage of germinated conidia on each plate was assessed every 4 h by counting the number of germinated conidia under the microscope until 24 h.

### Stress tolerance assays

For chemical stress assays, conidia (1 µL from 1 × 10^6^ conidia/mL suspension) were inoculated at the centre of SDAY plates supplemented with NaCl (0.5 M), Congo red (1 mg/mL) or H_2_O_2_ (3 mM), and radial growth (colony diameter) was measured after a 10 d incubation [81].

### Virulence assays

For virulence assays on *G. mellonella* larvae, cuticular infection and intrahaemocoelic injection were conducted according to previously described methods [74,82]. For cuticular infection, *G. mellonella* larvae were immersed for 90 s in 50 mL aliquots of 1 × 10^7^ conidia/mL suspension (treatments) or water (control), and then transferred onto paper to remove excess water. For injection, 1 μL of 1 × 10^5^ conidia/mL suspension (treatments) or water (control) was injected into the haemocoel of each larva. Mortality was recorded every 12 h after treatment and dead larvae were further incubated at 25 °C after surface sterilization. The assay was performed in triplicate, with 25 insects per replicate for each condition.

### Conidial hydrophobicity, adherence and penetration assays

Hydrophobicity assays were performed as described previously [83]. Liquid paraffin (300 μL) was added into 3 mL of 1 × 10^7^ conidia/mL suspension. The mixture was vortexed for 2 min in a 20 mL standard separation funnel and the organic phase was allowed to separate from the aqueous phase. Subsequently, conidia in the aqueous phase were counted under the microscope using Neubauer hemocytometers. The assay was performed in triplicate, with 3 measurements per strain.

Conidial adherence assays were performed as described previously [84]. Hind wings of Asian migratory locusts (*Locusta migratoria manilensis*), sterilized in 30% (v/v) H_2_O_2_ for 5 min, were gently floated on the surface of each suspension for 30 s and then placed on 0.7% (w/v) water agar with the conidial attachment side facing up. Following incubation for 8 h, the number of conidia adhering to the surface was counted under a microscope both before and after washing the samples with sterile water for 30 s, during which unattached conidia were removed using a pipette tip. The assay was conducted in triplicate, with three measurements were taken from each wing for every strain.

For penetration assays, 1 μL conidia suspension (1 × 10^5^ conidia/mL) with 0.05% (v/v) Tween 80 was inoculated on the surface of cicada wings placed on SDAY plates. After 3 d, the wings were removed, the SDAY plates were incubated for another 3 d to measure the colony diameter for each strain [85].

### Quantification and statistical analysis

All data were analyzed using GraphPad Prism version 7.0 and SPSS v23.0 (SPSS Inc., Chicago, IL, USA). The median lethal time (LT_50_) for insect larvae was calculated using SPSS 23.0. Kolmogorov-Smirnov test and Levene’s test were used to confirm that the data followed normality and homoscedasticity, respectively. Subsequently, data (mean ± SD) from different experimental groups were analyzed using Student’s t-test, one-way analysis of variance (ANOVA) followed by a least significant difference (LSD) test. *P*-values of 0.05 or lower were considered statistically significant.

## Supporting information

S1 Fig Characteristics of BbPmV4-2. (A) Comparison of the 5′- and 3′-termini from BbPmV4-2 dsRNA sequences. Asterisks (*) indicate conserved nucleotides. (B) The phylogenetic tree based on RdRp constructed by the maximum-likelihood (ML) method using the LG + G + F amino acid substitution model. The scale bar represents 0.5 amino acid substitutions per site, and numbers at the nodes indicate bootstrap support over 50% (1000 replicates).
(TIF)

S2 Fig Construction of VI and VF isogenic lines. (A) Agarose gel electrophoresis of purified dsRNA. M indicates the DNA marker whose sizes are shown on the left; lanes 2151-VI 1/2/3 contain extracts from VI strains; lanes 2151-VF 1/2/3 contain extracts from VI strains. (B) Agarose gel electrophoresis of RT-PCR amplicons from total RNA using primers specific for BbPmV4-2 ORF1 and ORF5. M indicates the DNA marker whose sizes are shown on the right; lanes 2151-VI 1/2/3 contain RT-PCR amplicons from VI strains; lanes 2151-VF 1/2/3 contain RT-PCR amplicons from VI strains. (C) Agarose gel electrophoresis of RT-PCR amplicons from dsRNA using primers specific for BbPmV4-2 ORF1 and ORF5. M indicates the DNA marker whose sizes are shown on the right; lanes 2151-VI 1/2/3 contain RT-PCR amplicons from VI strains; lanes 2151-VF 1/2/3 contain RT-PCR amplicons from VI strains.
(TIF)

S3 Fig Impacts of BbPmV4-2 on growth, conidiation, and chemical stress responses. (A) Colony morphology of VF and VI strains (25 °C, 10 d). (B) Conidial yields of VF and VI strains at 7 d, 10 d, or 14 d. (C) Relative expression levels in VF and VI strains of eight conidiation related genes 3 dpi, as measured by RT-qPCR. For each gene, expression in VF was set as 1 and relative expression in VI was calculated. (D) and (E) Colony morphology and diameters of VF and VI strains on SDAY, 1/4SDAY and PDA plates 10 dpi. (F) and (G) Colony morphology and relative growth inhibition of VF and VI strains on SDAY plates containing NaCl, Congo red, and H_2_O_2_. Data were present as the mean ± standard deviation (SD) from three replications. * *P* < 0.05, ** *P* < 0.01 or *** *P* < 0.001.
(TIF)

S4 Fig Expression of BbPmV4-2 ORFs 1–8 in VF strain. (A) Agarose gel electrophoresis of PCR amplicons using specific primers for BbPmV4-2 ORFs 1–8. (B) Agarose gel electrophoresis of RT-PCR amplicons using specific primers for BbPmV4-2 ORFs 1–8. (C) Relative expression levels in VF and VI strains of eight ORFs, as measured by RT-qPCR. For each ORF, expression in VI was set as 1 and relative expression in VF was calculated. (D) Immunoblotting of BbPmV4-2 ORFs 1–5 and ORFs 7–8 encoded proteins. Data were present as the mean ± standard deviation (SD) from three replications. * *P* < 0.05, ** *P* < 0.01 or *** *P* < 0.001.
(TIF)

S5 Fig Bioinformatics analysis of BbGAP1 and related proteins. (A) Phylogenetic analysis and (B) structure domain analysis of BbGAP1. (C) LSCM images (scale bars: 1 μm) of the BbGAP1 subcellular location, showing that BbGAP1 is localized in the *B.bassiana*, with the GFP signal apparent in spores and hyphae. The white arrows indicate localization on the cell membrane, while the red arrows denote cytoplasmic localization.
(TIF)

S6 Fig *BbGap1* deletion in *B. bassiana.* (A) Schematic diagram of the *BbGap1* deletion strategy by homologous recombination. (B) *BbGap1* validation (part of *BbGap1* coding region) and (C) Bar validation by PCR confirmation. Genomic DNAs extracted from different strains were used as templates for PCR. M, Marker, RT-PCR verification of (D) *BbAct* and (E) *BbGap1* in VF and *ΔBbGap1* strains. The expression of *BbAct* as the control and *BbGap1* was used for RT-PCR confirmation with cDNA as a template.
(TIF)

S7 Fig Effect of *BbGap1* deletion on *B. bassiana.* (A) Survival of *G. mellonella* larvae and (B) LT_50_ after injection with *ΔBbGap1* strains. CK larvae were treated with sterile water. Student’s t-test or ANOVA, * *P* < 0.05, ** *P* < 0.01 or *** *P* < 0.001. Similar results were obtained for three biological replicates.
(TIF)

S8 Fig Bioinformatics analysis of BbSDU1 and related proteins. (A) Phylogenetic analysis and (B) structure domain analysis of BbSDU1. (C) LSCM images (scale bars: 1 μm) of the BbSDU1 subcellular location, showing that BbSDU1 is localized in the cell vacuole. This was confirmed using the vacuole-specific dye CMAC (7-amino-4-chloromethylcoumarin), a blue-fluorescent vital dye that selectively accumulates in acidic vacuolar compartments.
(TIF)

S9 Fig *BbSdu1* deletion in *B. bassiana.* (A) Schematic diagram of the *BbSdu1* deletion strategy by homologous recombination. (B) *BbSdu1* validation (part of *BbSdu1* coding region), (C) Bar, and (D) Ben validation by PCR confirmation. Genomic DNAs extracted from different strains were used as templates for PCR. M, Marker, RT-PCR verification of (E) *BbAct* and (F) *BbSdu1* in VF, *ΔBbSdu1* and CP strains. The expression of *BbAct* as the control and *BbSdu1* was used for RT-PCR confirmation with cDNA as a template.
(TIF)

S10 Fig Virulence of *BbSdu1* deletion on *B. bassiana.* (A) Survival of *G. mellonella* larvae and (B) LT_50_ after injection with *ΔBbSdu1* strains. CK larvae were treated with sterile water. (C) Relative expression levels in VF and VI strains of five hydrophobicity genes and three conidial adherence genes, as measured by RT-qPCR. (D) Relative expression levels in VF and *ΔBbSdu1* strains of eight cuticle degradation and virulence genes, as measured by RT-qPCR. For each gene, expression in VF was set as 1 and relative expression in VI was calculated. Student’s t-test or ANOVA, * *P* < 0.05, ** *P* < 0.01 or *** *P* < 0.001. Similar results were obtained for three biological replicates.
(TIF)

S11 Fig Effects of P5 on ubiquitination levels of total proteins. (A) The ubiquitination levels of total proteins were measured by an anti-ubiquitin antibody. *VF-ORF5*^*oe*^ (Expressing ORF5 in the VF strain) and *VI-ORF5*^*RNAi*^ (interference with ORF5 expression in VI strain).
(TIF)

S1 Table Paired primers used for RT-PCR of BbPmV4-2. (DOCX)

S2 Table Paired primers used for RT-qPCR in *B. bassiana.* (DOCX)

S3 Table Results of Y2H screening. (DOCX)

S4 Table Paired primers used for PCR in the Y2H, BiFC, and Co-IP assays. (DOCX)

S5 Table Paired primers used for PCR, RT-PCR, and RT-qPCR of ORF1–8. (DOCX)

S6 Table Paired primers used for PCR of ORF1–8 + FLAG. (DOCX)

S7 Table Paired primers used for manipulation of target genes in Bb2151. (DOCX)
