# Supplementary figures and images for: A mycovirus enhances fitness of an insect pathogenic fungus and potentially modulates virulence through interactions between viral and host proteins
Source: PLoS Pathog. 2025 Oct 23;21(10):e1013634. doi: 10.1371/journal.ppat.1013634 (PMC12574890; doi:10.1371/journal.ppat.1013634)

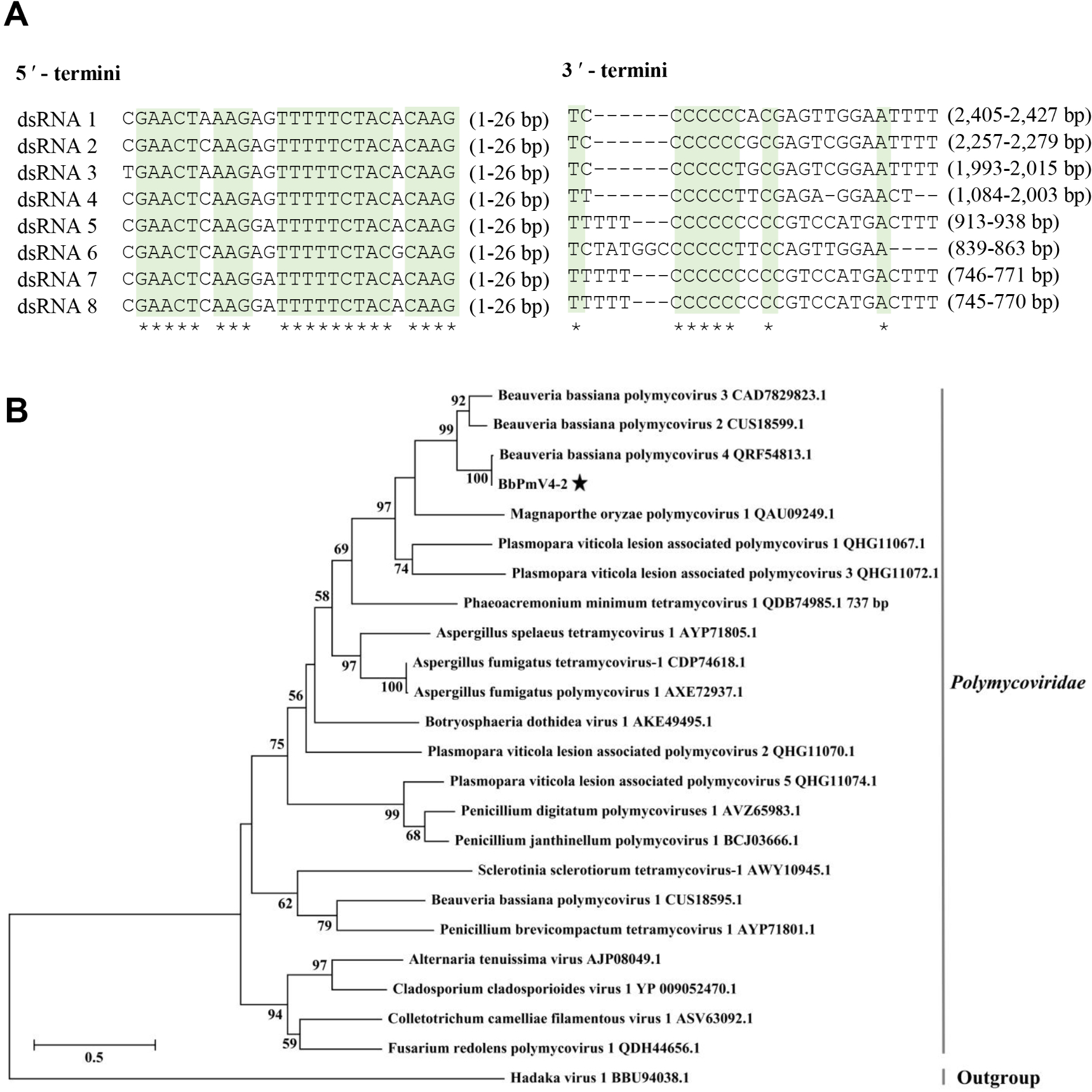

Supplement: S1 Fig — (A) Comparison of the 5′- and 3′-termini from BbPmV4-2 dsRNA sequences. Asterisks (*) indicate conserved nucleotides. (B) The phylogenetic tree based on RdRp constructed by the maximum-likelihood (ML) method using the LG + G + F amino acid substitution model. The scale bar represents 0.5 amino acid substitutions per site, and numbers at the nodes indicate bootstrap support over 50% (1000 replicates). (TIF) [file ppat.1013634.s001.tif]

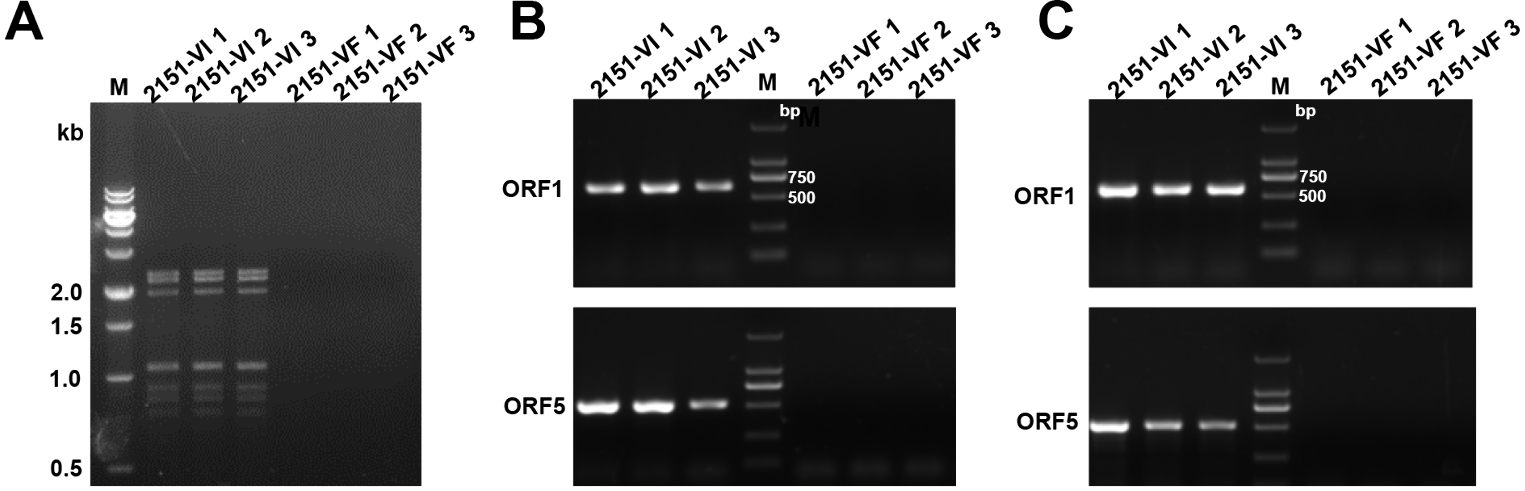

Supplement: S2 Fig — (A) Agarose gel electrophoresis of purified dsRNA. M indicates the DNA marker whose sizes are shown on the left; lanes 2151-VI 1/2/3 contain extracts from VI strains; lanes 2151-VF 1/2/3 contain extracts from VI strains. (B) Agarose gel electrophoresis of RT-PCR amplicons from total RNA using primers specific for BbPmV4-2 ORF1 and ORF5. M indicates the DNA marker whose sizes are shown on the right; lanes 2151-VI 1/2/3 contain RT-PCR amplicons from VI strains; lanes 2151-VF 1/2/3 contain RT-PCR amplicons from VI strains. (C) Agarose gel electrophoresis of RT-PCR amplicons from dsRNA using primers specific for BbPmV4-2 ORF1 and ORF5. M indicates the DNA marker whose sizes are shown on the right; lanes 2151-VI 1/2/3 contain RT-PCR amplicons from VI strains; lanes 2151-VF 1/2/3 contain RT-PCR amplicons from VI strains. (TIF) [file ppat.1013634.s002.tif]

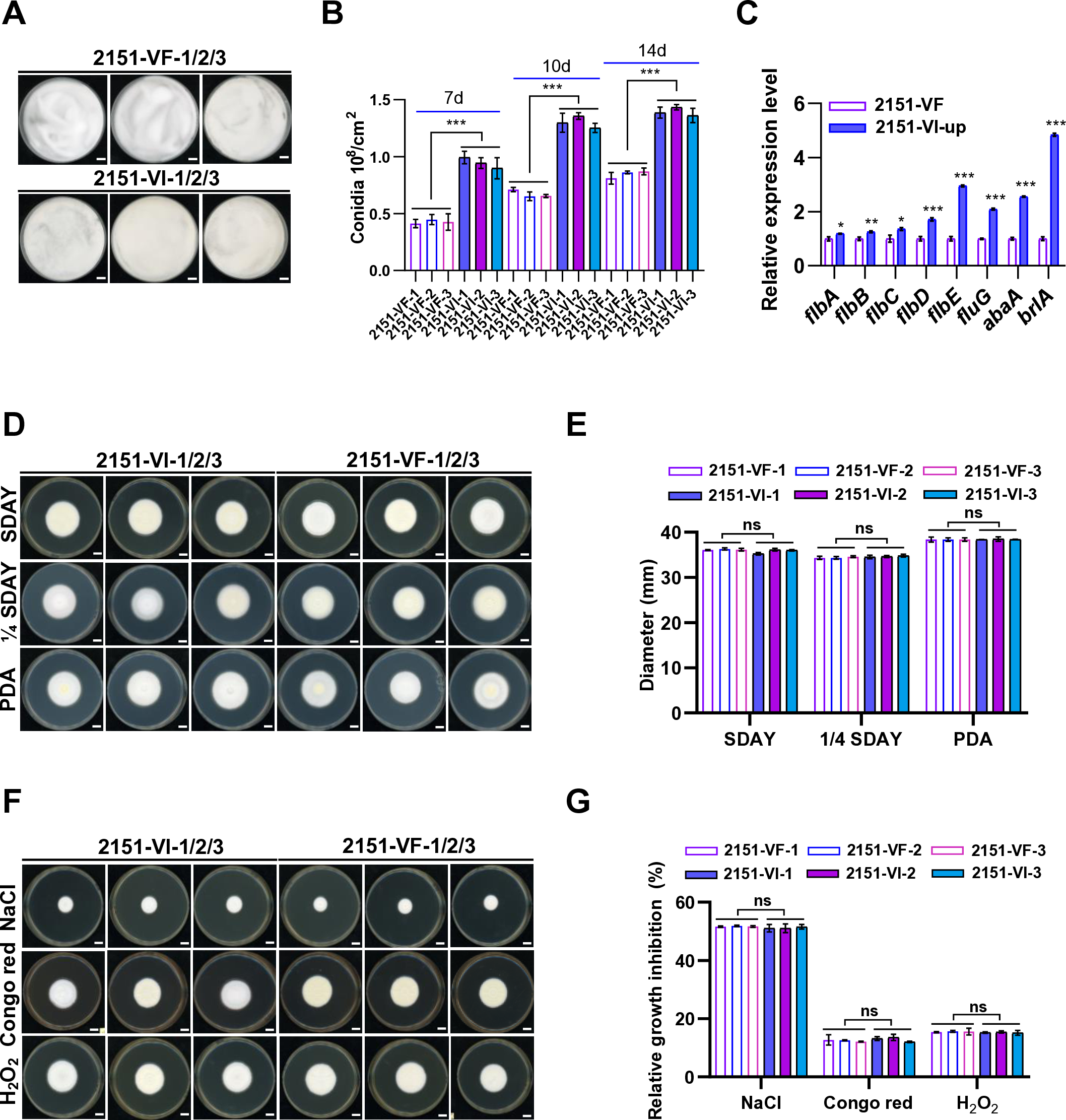

Supplement: S3 Fig — (A) Colony morphology of VF and VI strains (25 °C, 10 d). (B) Conidial yields of VF and VI strains at 7 d, 10 d, or 14 d. (C) Relative expression levels in VF and VI strains of eight conidiation related genes 3 dpi, as measured by RT-qPCR. For each gene, expression in VF was set as 1 and relative expression in VI was calculated. (D) and (E) Colony morphology and diameters of VF and VI strains on SDAY, 1/4SDAY and PDA plates 10 dpi. (F) and (G) Colony morphology and relative growth inhibition of VF and VI strains on SDAY plates containing NaCl, Congo red, and H2O2. Data were present as the mean ± standard deviation (SD) from three replications. * P < 0.05, ** P < 0.01 or *** P < 0.001. (TIF) [file ppat.1013634.s003.tif]

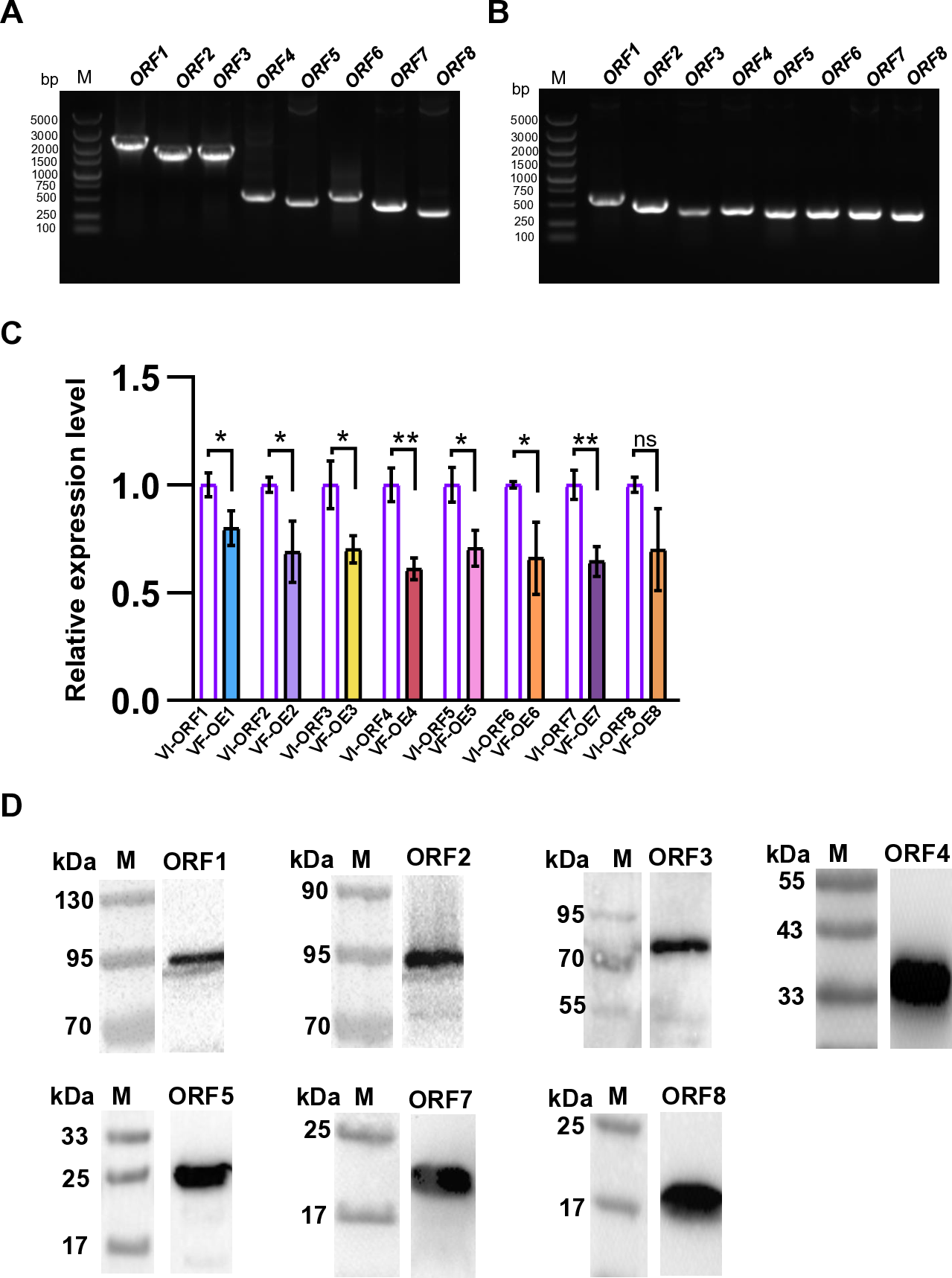

Supplement: S4 Fig — (A) Agarose gel electrophoresis of PCR amplicons using specific primers for BbPmV4-2 ORFs 1–8. (B) Agarose gel electrophoresis of RT-PCR amplicons using specific primers for BbPmV4-2 ORFs 1–8. (C) Relative expression levels in VF and VI strains of eight ORFs, as measured by RT-qPCR. For each ORF, expression in VI was set as 1 and relative expression in VF was calculated. (D) Immunoblotting of BbPmV4-2 ORFs 1–5 and ORFs 7–8 encoded proteins. Data were present as the mean ± standard deviation (SD) from three replications. * P < 0.05, ** P < 0.01 or *** P < 0.001. (TIF) [file ppat.1013634.s004.tif]

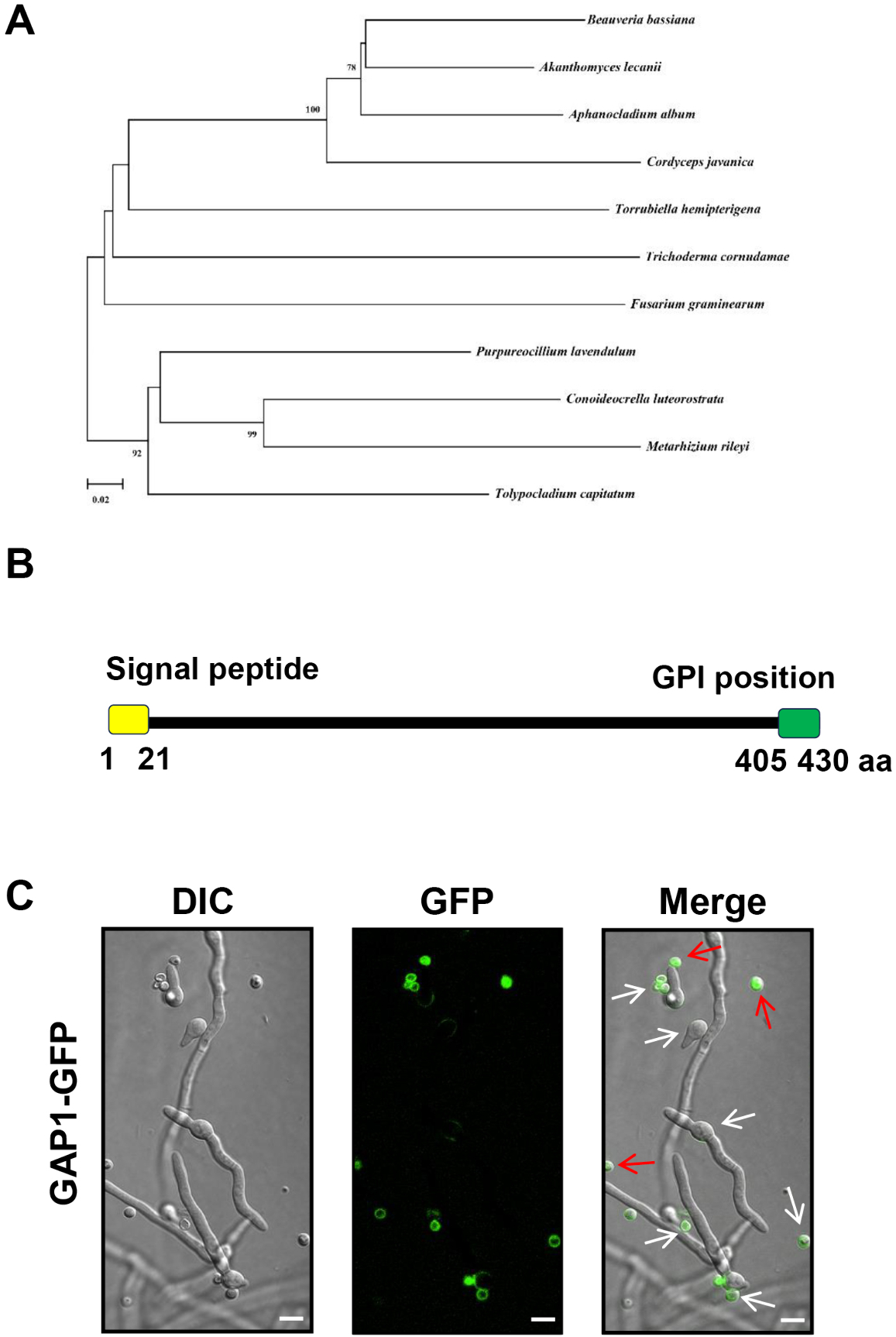

Supplement: S5 Fig — (A) Phylogenetic analysis and (B) structure domain analysis of BbGAP1. (C) LSCM images (scale bars: 1 μm) of the BbGAP1 subcellular location, showing that BbGAP1 is localized in the B.bassiana, with the GFP signal apparent in spores and hyphae. The white arrows indicate localization on the cell membrane, while the red arrows denote cytoplasmic localization. (TIF) [file ppat.1013634.s005.tif]

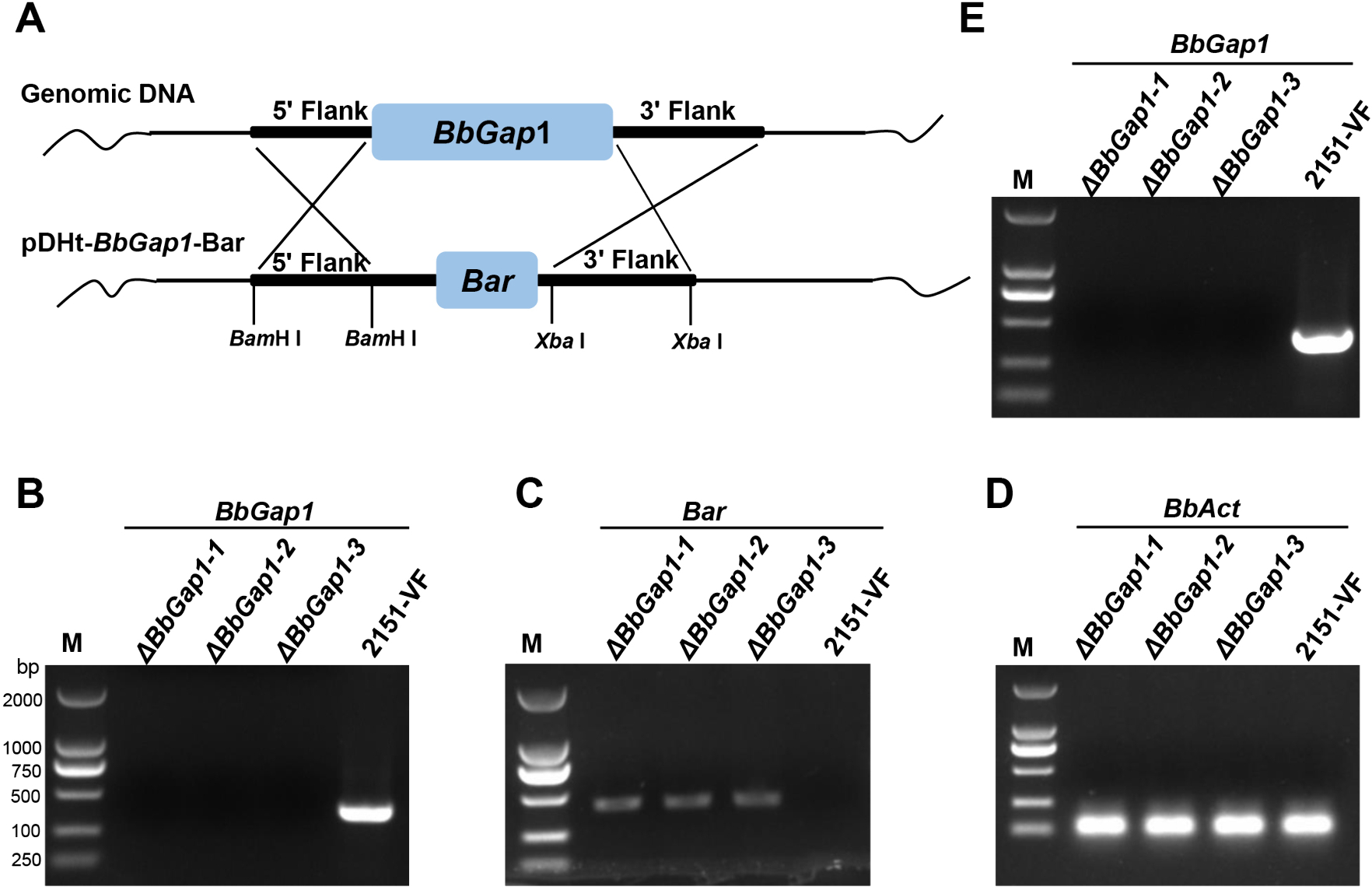

Supplement: S6 Fig — (A) Schematic diagram of the BbGap1 deletion strategy by homologous recombination. (B) BbGap1 validation (part of BbGap1 coding region) and (C) Bar validation by PCR confirmation. Genomic DNAs extracted from different strains were used as templates for PCR. M, Marker, RT-PCR verification of (D) BbAct and (E) BbGap1 in VF and ΔBbGap1 strains. The expression of BbAct as the control and BbGap1 was used for RT-PCR confirmation with cDNA as a template. (TIF) [file ppat.1013634.s006.tif]

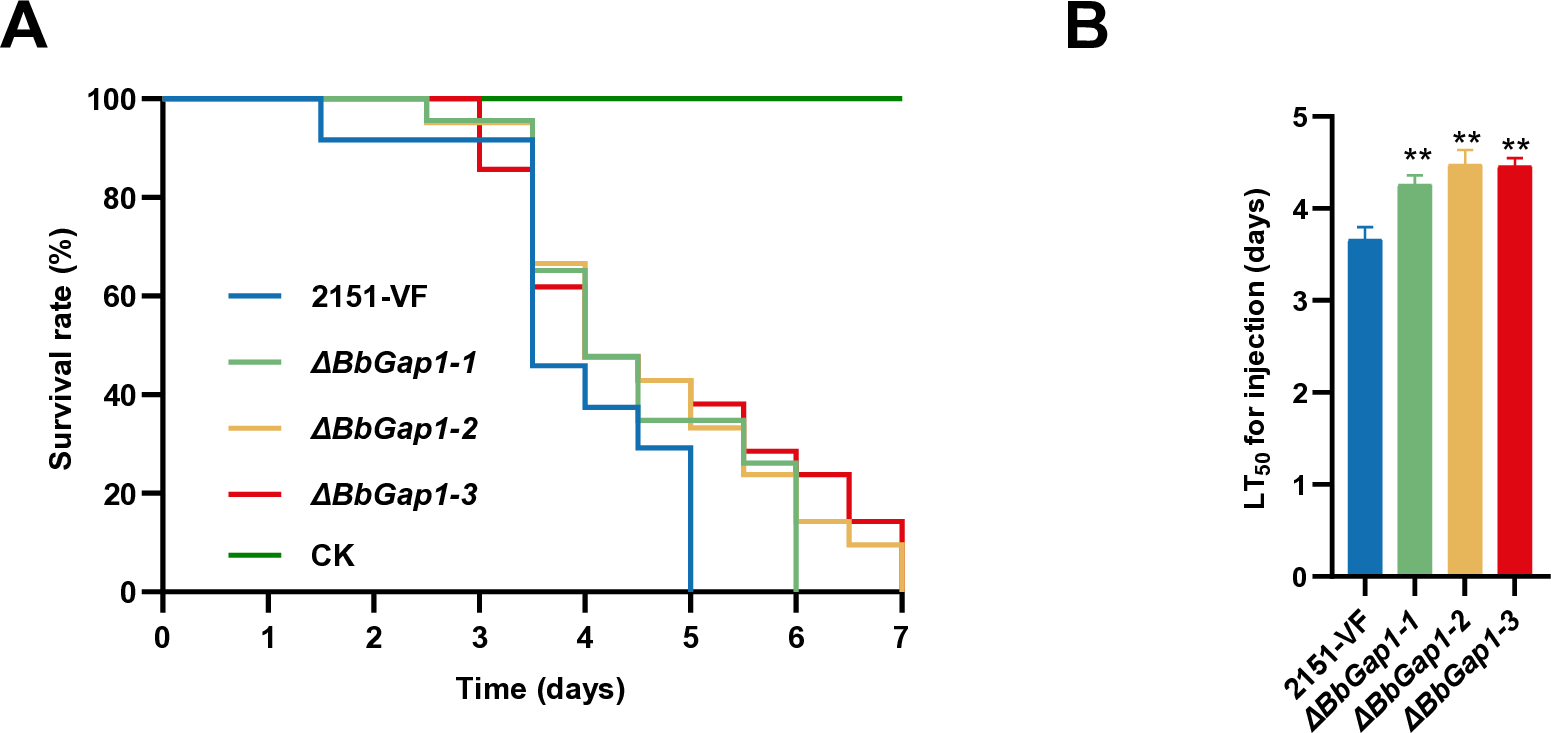

Supplement: S7 Fig — (A) Survival of G. mellonella larvae and (B) LT50 after injection with ΔBbGap1 strains. CK larvae were treated with sterile water. Student’s t-test or ANOVA, * P < 0.05, ** P < 0.01 or *** P < 0.001. Similar results were obtained for three biological replicates. (TIF) [file ppat.1013634.s007.tif]

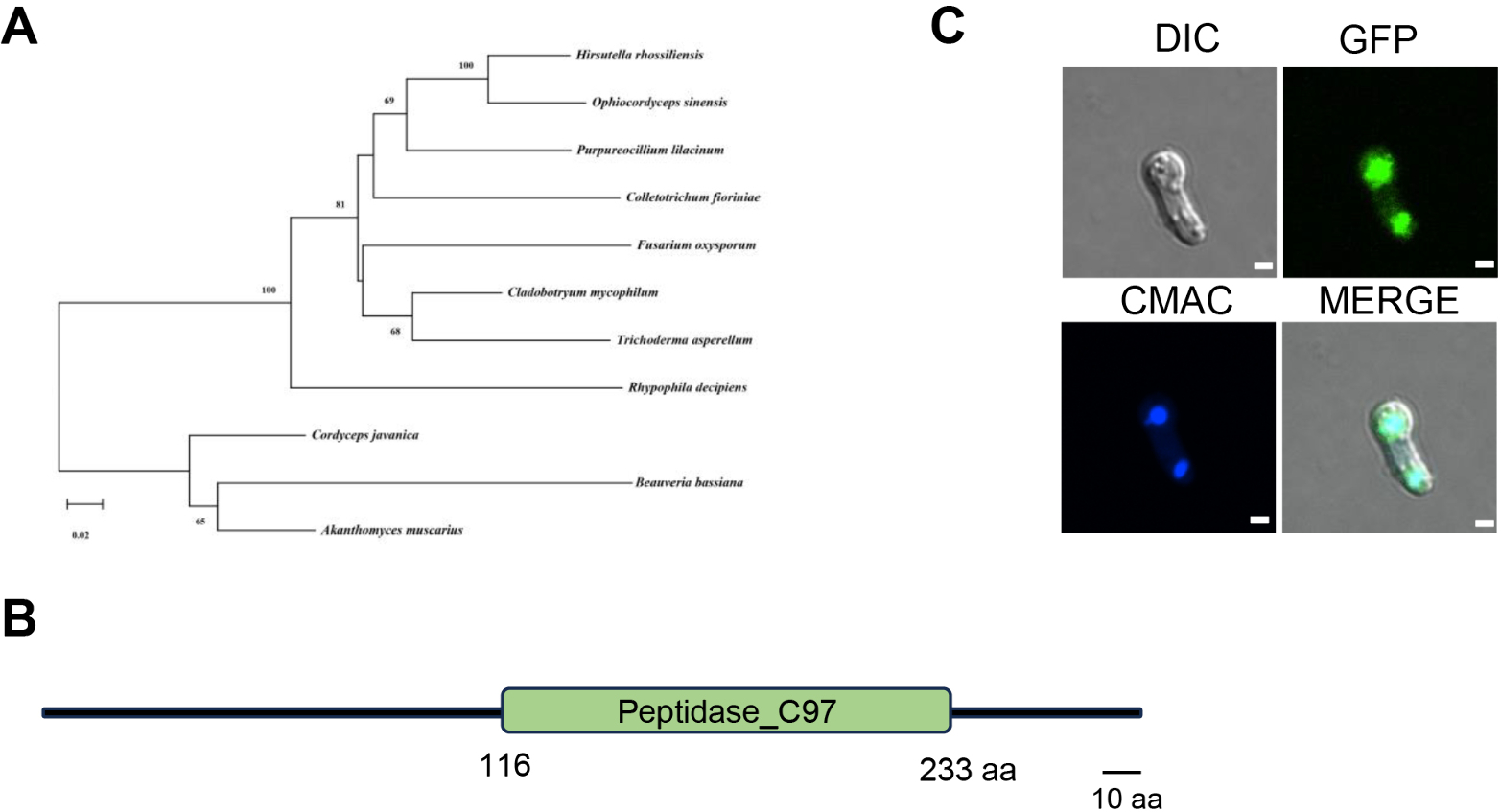

Supplement: S8 Fig — (A) Phylogenetic analysis and (B) structure domain analysis of BbSDU1. (C) LSCM images (scale bars: 1 μm) of the BbSDU1 subcellular location, showing that BbSDU1 is localized in the cell vacuole. This was confirmed using the vacuole-specific dye CMAC (7-amino-4-chloromethylcoumarin), a blue-fluorescent vital dye that selectively accumulates in acidic vacuolar compartments. (TIF) [file ppat.1013634.s008.tif]

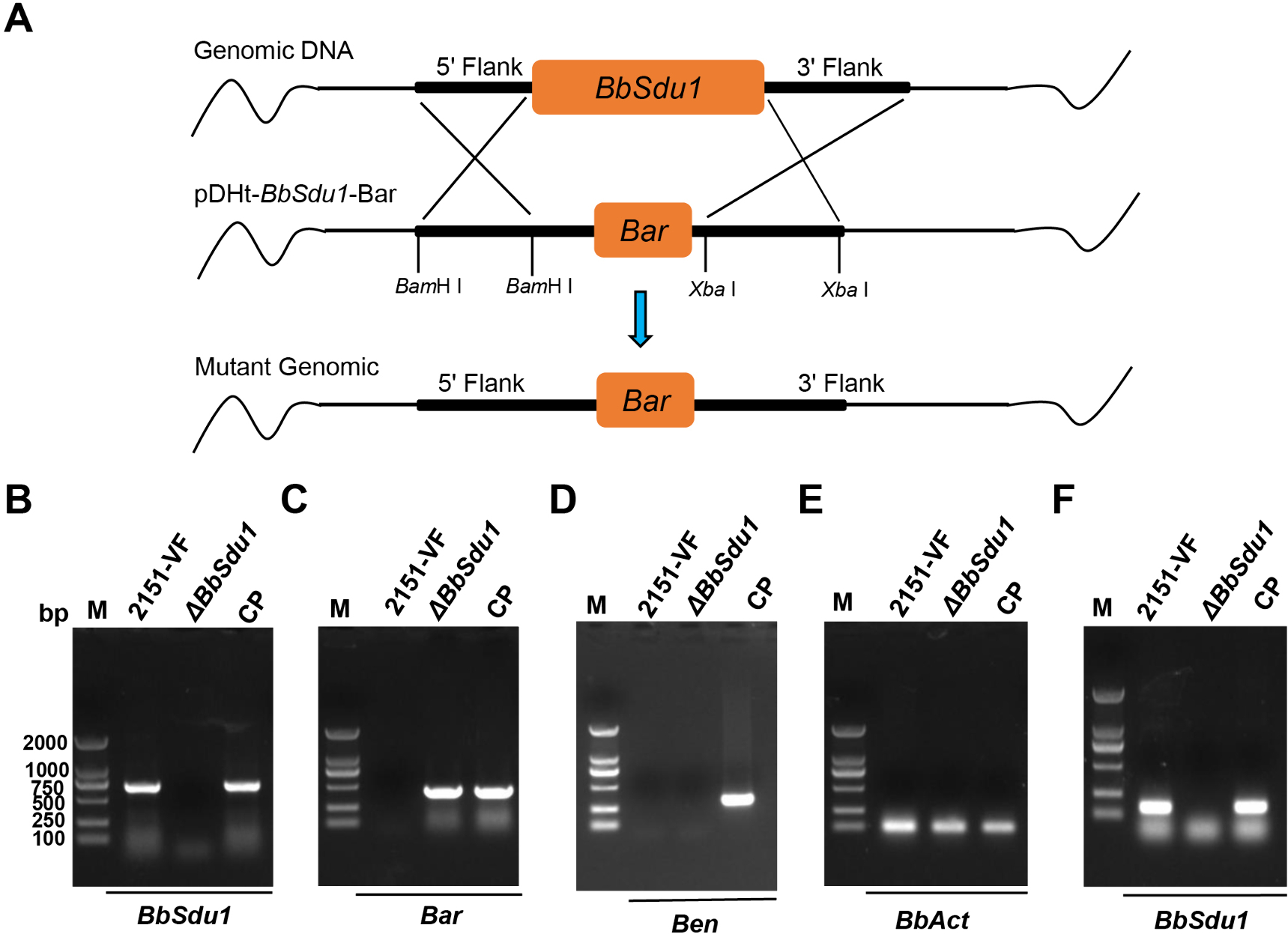

Supplement: S9 Fig — (A) Schematic diagram of the BbSdu1 deletion strategy by homologous recombination. (B) BbSdu1 validation (part of BbSdu1 coding region), (C) Bar, and (D) Ben validation by PCR confirmation. Genomic DNAs extracted from different strains were used as templates for PCR. M, Marker, RT-PCR verification of (E) BbAct and (F) BbSdu1 in VF, ΔBbSdu1 and CP strains. The expression of BbAct as the control and BbSdu1 was used for RT-PCR confirmation with cDNA as a template. (TIF) [file ppat.1013634.s009.tif]

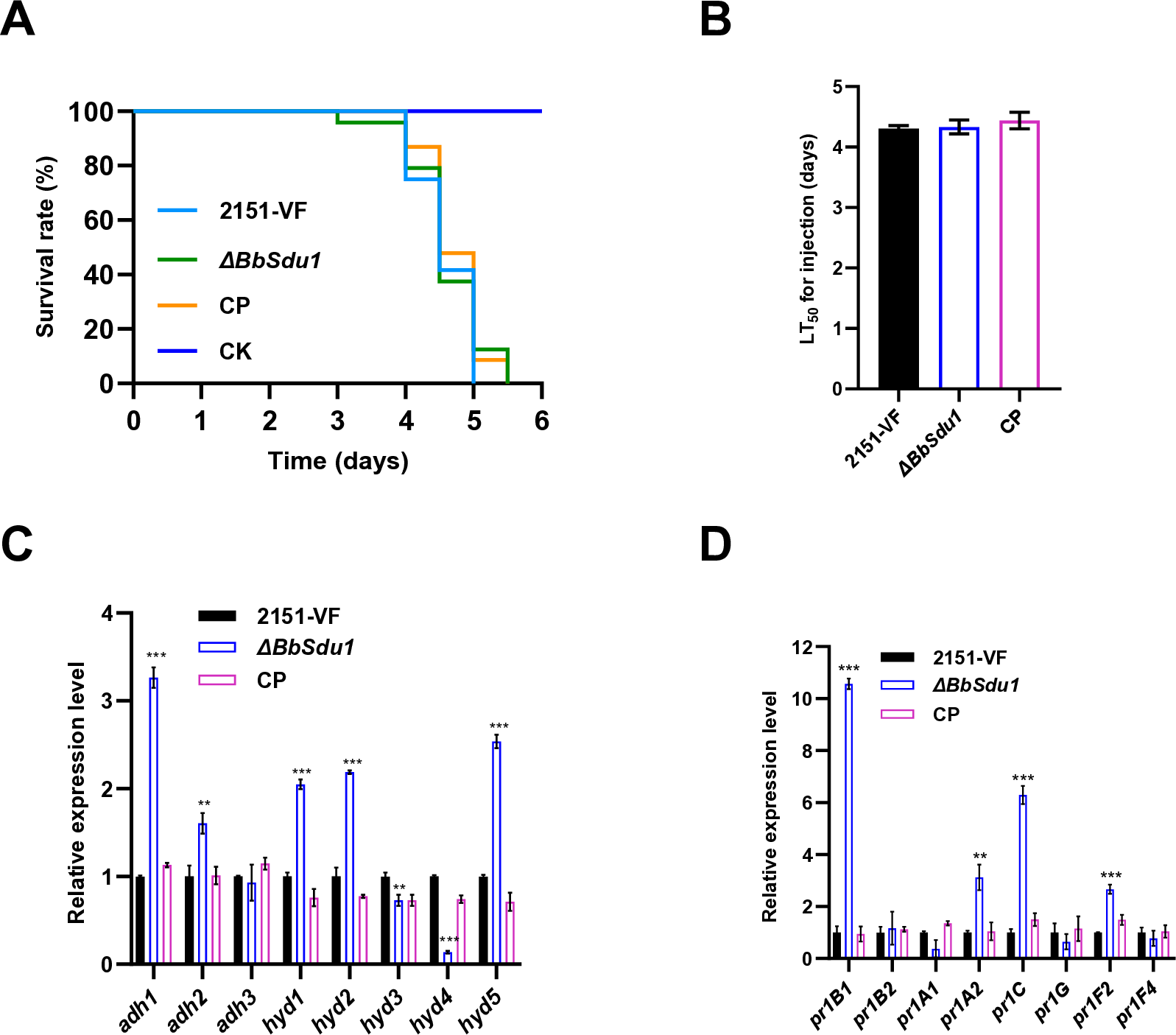

Supplement: S10 Fig — (A) Survival of G. mellonella larvae and (B) LT50 after injection with ΔBbSdu1 strains. CK larvae were treated with sterile water. (C) Relative expression levels in VF and VI strains of five hydrophobicity genes and three conidial adherence genes, as measured by RT-qPCR. (D) Relative expression levels in VF and ΔBbSdu1 strains of eight cuticle degradation and virulence genes, as measured by RT-qPCR. For each gene, expression in VF was set as 1 and relative expression in VI was calculated. Student’s t-test or ANOVA, * P < 0.05, ** P < 0.01 or *** P < 0.001. Similar results were obtained for three biological replicates. (TIF) [file ppat.1013634.s010.tif]

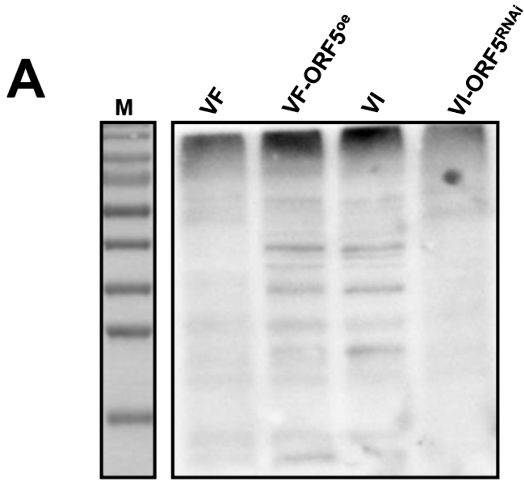

Supplement: S11 Fig — (A) The ubiquitination levels of total proteins were measured by an anti-ubiquitin antibody. VF-ORF5oe (Expressing ORF5 in the VF strain) and VI-ORF5RNAi (interference with ORF5 expression in VI strain). (TIF) [file ppat.1013634.s011.tif]
