# Supplementary material for: A mycovirus enhances fitness of an insect pathogenic fungus and potentially modulates virulence through interactions between viral and host proteins
Source: PLoS Pathog. 2025 Oct 23;21(10):e1013634. doi: 10.1371/journal.ppat.1013634 (PMC12574890; doi:10.1371/journal.ppat.1013634)
Supplement: S1 Table — (DOCX) [file ppat.1013634.s012.docx]

**S1 Table.** Paired primers used for RT-PCR of BbPmV4-2.

| Primers | Sequences (5′ to 3′) | Products |
| --- | --- | --- |
| ORF1-F/ORF1-R | GTCACCGATAGGAAAGACCA/  CAAGGAGCTTCTCGTTGAGC | 574 bp |
| ORF5-F/ORF5-R | CAATCCAGCGTTTTCGTTGGTG/  CCTCGATAGCCAACACATCAAC | 519 bp |
| D7F/D7R | ATGCCTTTTCTTGGCACCCAC/  CGGGCCCAGGGTCGATGACA | 316 bp (dsRNA5)  316 bp (dsRNA 7)  148 bp (dsRNA 8) |
| D8F/D8R | TGTCATCGACCCTGGGCCCG/  AGGCAGCCCTCGATAGCCAAC | 314 bp (dsRNA 5)  147 bp (dsRNA 7)  314 bp (dsRNA 8) |
|  |  |  |
