# Supplementary material for: A mycovirus enhances fitness of an insect pathogenic fungus and potentially modulates virulence through interactions between viral and host proteins
Source: PLoS Pathog. 2025 Oct 23;21(10):e1013634. doi: 10.1371/journal.ppat.1013634 (PMC12574890; doi:10.1371/journal.ppat.1013634)
Supplement: S2 Table — (DOCX) [file ppat.1013634.s013.docx]

**S2 Table.** Paired primers used for RT-qPCR in *B. bassiana*.

| Gene | Tag locus* | | | Annotation | Sequences (5′ to 3′) of paired primers | |  |  |
| --- | --- | --- | --- | --- | --- | --- | --- | --- |
| **Used as reference genes** | | | | | | |  |  |
| ACT | BBA_04860 | | | β-actin | CCATCAACCCCAAGTCCAACC/  GCCAGAGGCGTAGAGGGAGAG | |  |  |
| **Involved in asexual development** | | | | | | |  |  |
| *fluG* | BBA_04942 | | | Developmental protein FluG | CTATGCCAGCGTCATGTGC/ CTCAAATTCGACTTCAAAAC | |  |  |
| *flbA* | BBA_02968 | | | Regulator of G protein signaling | GGTGTCAAGATGGCTGCTGA/ GCAATCTCGATGGTCTCGCG | |  |  |
| *flbB* | BBA_06988 | | | BZIP-type transcription factor | CGATCTCGATGGCGAGAT/ CAATTCAAAATCCTCCAT | |  |  |
| *flbC* | BBA_03181 | | | putative zinc finger protein | TTGCCCGACTTGCAACAA/ TCATGTTGCTGCGCACGC | |  |  |
| *flbD* | BBA_07259 | | | myb-like DNA-binding domain-containing protein | GCAATGCCGCGAGCGCTTTC/ CGATTCTGGCTGCCGTTCCA | |  |  |
| *flbE* | BBA_01716 | | | AorFlbE-like protein | TACGACCCGGACGAGACGGTCA/ ACCACCACGTACCAGCCAATG | |  |  |
| *brlA* | BBA_07544 | | | zinc finger protein odd-paired-like (opl) | CACCGCAAGATGATGGTGCA/ CGGGGTAGTCGCACTTGC | |  |  |
| *abaA* | BBA_00300 | | | FoabaA-like protein | AGTTCTTTGAGGACCTGCGC/ GTCATAGGCAATCTCGTCGC | |  |  |
| **Involved in DNA damage repair** | | | | | | |  |  |
| *asf1* | BBA_01428 | | | ASF1 like histone chaperone | ATCAAGAGCTCGACTCCCTC/ TGAGCTCAGGAGAGTCGTAT | |  |  |
| *chk2* | BBA_06016 | | | checkpoint kinase 2 | ACTAATGACGCGACATACGG/ GCAAAATCGAGATGCCTGT | |  |  |
| *mec1* | BBA_02334 | | | Putative protein kinase MEC1 | AGCTGTACATTCGCACCTAC/ GAAGATGCGAATCTTGCTCT | |  |  |
| *rad52* | BBA_06117 | | | Putative recombinase  RAD52 | CACTGCGCAGCTTTGGCAAT/ CAGAACTAGACCCTGACGCT | |  |  |
| *rad53* | BBA_00126 | | | putative protein kinase RAD53 | ACCAGGACACGACACAGAT/ CGCATCTCATTTTCCACCTT | |  |  |
| *tel1* | BBA_02241 | | | PDNA-binding protein kinase TEL1 | CTGTCGCCAATATCTGGCT/ CTCGAATCTAGCTTTGCAAT | |  |  |
| *yku70* | BBA_06892 | | | ku70 protein | AGCGGTCGCTATTCTCTAAT/ GACGGTTCGCGTACTATCT | |  |  |
| *top1* | BBA_00975 | | | topoisomerase I | ATATCAGCAGTGGCGTACCA/ TCGCTTCTCGTTATCCTTCT | |  |  |
| **Involved in heat shock** | | | | | | |  |  |
| *hsp20* | BBA_07886 | | | hsp20-like protein | GCCTTCTTTGAACAGATGATG/ AGTCGCCATCATCTACAGTC | |  |  |
|  |  | | |  |  | |  |  |
| **S2 table.** (continued) | | | | | | |  | |
| Gene | | | Tag locus* | Annotation | | | Sequences (5′ to 3′) of paired primers | |
| *hsp30b* | | | BBA_02057 | heat shock protein 30 | | | ACGAGTTTGACACATACTCG/ ACGCTCACGTCATTCTTGTTG | |
| *hsp40a* | | | BBA_06930 | Chaperone DnaJ | | | TGCTAAAATCTACGGCGCC/ GCTTGTTCGTCGTGTGAAAT | |
| *hsp40c* | | | BBA_03736 | chaperone protein dnaJ 2 | | | AGGCCAGGTAGAGATTGACC/ TCATCGGCGAGAATCTCGT | |
| *hsp60* | | | BBA_05467 | chaperonin GroL | | | AGCTTCCACCAAGTTCCGAG/ TCCTTCAAGGTAACAGCACG | |
| *hsp70a* | | | BBA_00941 | hsp70 protein | | | AGATGGTGCCATGGGCAA/ TTGAGAATGGTGCTGTC | |
| *hsp90* | | | BBA_06516 | heat shock protein Hsp90 | | | ATCATCAACACCGTCTACTC/ GGCAATCGTACCAAGGTTGT | |
| *hsp104* | | | BBA_02283 | heat shock protein HSP98 | | | ATGACGGCACGAATGGAT/ CAGACGAACCAAGAGCTTCT | |
| **Involved in cuticle degradation and virulence** | | | | | | | | |
| *pr1B 1* | | | BBA_00443 | cuticle-degrading protease bassiasin I precursor | | | GCTCAGCGGCGTCATTG/ TGGGCGGCATCCCTATT | |
| *pr1B 2* | | | BBA_09500 | cuticle-degrading serine protease | | | ACATTGTCAAGCTCAAGGACAC/ AACGGCGTTGATGCTAACAA | |
| *pr1A1* | | | BBA_04617 | subtilisin-like protease Pr1A | | | ATCATTGCCGGCATGGACTA/ TGCAGACAGTCTCCTCCGA | |
| *pr1A2* | | | BBA_03653 | subtilisin-like protease Pr1B | | | ATGGCGAATCTGAGCATC/ TCGCCACGCAGACTTGAT | |
| *pr1C* | | | BBA_09153 | subtilisin-like serine protease PR1C | | | GCCGATGAAAAGGCCAAGA/ TGTAACCCTTGGCACGCA | |
| *pr1F2* | | | BBA_07143 | subtilisin-like protease PR1F | | | AGGCCGTCATCAACCTTTC/ GGCCCGAATATATCCACTGT | |
| *pr1F4* | | | BBA_07320 | subtilisin-like protease PR1F | | | GAAATTCACCAGTACCTTTGC/ AATCCGCCGTCCAGAGGTTCT | |
| *pr1G* | | | BBA_09270 | subtilisin-like protease PR1G | | | CTAGAGCCAAGCGATCCGA/ TCAAAATGGCCCGCGAAGCCT | |
| **Involved in conidial hydrophobicity** | | | | | | | | |
| *hyd1* | | | BBA_03015 | class I hydrophobin Hyd1 | | | CATGGTGGAAAGGATCTG/ ATCTTGGTCGTCTTCTCG | |
| *hyd2* | | | BBA_06599 | Class II hydrophobin Hyd2 | | | TTCTCAGCGATCTTGATCTT/ GCACTTGTTGTCGATTGG | |
| *hyd3* | | | BBA_00530 | Hydrophobin-like protein | | | GGCAATACCAGCTCCGGTAT/ TAGGAGAATTGCCGCCTGTA | |
| *hyd4* | | | BBA_03071 | hydrophobin-like protein | | | TACCGACGTCTCCTCCTTGT/ CTTCGCGCATATGTTCTTG | |
|  | | |  |  | | |  | |
| **S2 table** (continued) | | | | | | |  | |
| Gene | | | Tag locus* | Annotation | | | Sequences (5′ to 3′) of paired primers | |
| *hyd5* | | | BBA_02999 | hydrophobin-like protein | | | ACTCGCCATGCCTACCACT/ GCGTGGAGGCACAAGATTC | |
| **Involved in conidial adherence** | | | | | | | | |
| *adh1* | | | BBA_02419 | adhesin protein Mad1 | | | GTCAGCACCACCATCTGC/ GCAAGGAAGAGACTGCG | |
| *adh2* | | | BBA_02379 | adhesin protein Mad2 | | | TGGCACTACGTGCACCCG/ TGGTGGTGACGGTGGTGA | |
| *adh3* | | | BBA 03909 | filamentous hemagglutinin / adhesin | | | TACCACCTGTACAAGGTC/ TGGCGCTCAATGTAGCCG | |

* Gene accession codes of *B. bassiana* genome under the NCBI accession NL_ADAH00000000
