# Supplementary material for: A mycovirus enhances fitness of an insect pathogenic fungus and potentially modulates virulence through interactions between viral and host proteins
Source: PLoS Pathog. 2025 Oct 23;21(10):e1013634. doi: 10.1371/journal.ppat.1013634 (PMC12574890; doi:10.1371/journal.ppat.1013634)
Supplement: S3 Table — (DOCX) [file ppat.1013634.s014.docx]

**S3 Table.** Results of Y2H screening.

| Locus tag | Description | Protein_ID |
| --- | --- | --- |
| BBA_08688 | heat shock protein 30 | XP_008602007.1 |
| BBA_01741 | ThiJ/PfpI family protein | XP_008595060.1 |
| BBA_08237 | PPPDE peptidase family | XP_008601556.1 |
| BBA_06139 | GPI anchored protein | XP_008599458.1 |
| BBA_02268 | histone H4.1 | XP_008595587.1 |
| BBA_03627 | Hsp90 | XP_008596946.1 |
| BBA_04591 | glycerophosphoryl diester phosphodiesterase | XP_008597910.1 |
| BBA_08372 | uncharacterized protein | XP_008601691.1 |
| BBA_04032 | hypothetical protein | XP_008597351.1 |
| BBA_06766 | ATP synthase subunit E | XP_008600085.1 |
| BBA_02876 | vivid PAS protein VVD | XP_008596195.1 |
| BBA_02832 | elongation factor Tu GTP binding domain-containing protein | XP_008596151.1 |
| BBA_07201 | ER protein BIG1 | XP_008600520.1 |
| BBA_02283 | heat shock protein HSP98 | XP_008595602.1 |
| BBA_08336 | CAAX amino terminal protease | XP_008601655.1 |
| BBA_08152 | Protein of unknown function DUF1749 partial | XP_008601471.1 |
| BBA_01918 | hypothetical protein | XP_008595237.1 |
| BBA_09128 | Concanavalin A-like lectin/glucanase | XP_008602447.1 |
