# Supplementary material for: A mycovirus enhances fitness of an insect pathogenic fungus and potentially modulates virulence through interactions between viral and host proteins
Source: PLoS Pathog. 2025 Oct 23;21(10):e1013634. doi: 10.1371/journal.ppat.1013634 (PMC12574890; doi:10.1371/journal.ppat.1013634)
Supplement: S4 Table — (DOCX) [file ppat.1013634.s015.docx]

**S4 Table.** Paired primers used for PCR in the Y2H, BiFC, and Co-IP assays.

| Primers | Paired sequences (5'-3') | Purpose | Products |
| --- | --- | --- | --- |
| BD-ORF5_F/R | atggccatggaggccgaattcATGCCTTTTCTTGGCACCCA | Cloning and PCR detecting ORF5 | 513 bp |
|  | /ctagttatgcggccgctgcagTTACTGGCCAAAGACAGGGC |  |  |
| AD- *BbGap1* _F/R | gccatggaggccagtgaattcATGCTTCTCTTACCCCCATACTTG | Cloning and PCR detecting *BbGap1* | 1296 bp |
|  | /cagctcgagctcgatggatccTCATTGCACCATAACATTACAAGCA |  |  |
| ORF5+YVN_ P1_F/R | caagaacctttaatcgaattcATGCCTTTTCTTGGCACCCA | Cloning and PCR detecting ORF5 | 510 bp |
|  | /ttgctcaccatCTGGCCAAAGACAGGGCC |  |  |
| ORF5+YVN_ P2_F/R | ggccctgtctttggccagATGGTGAGCAA | Cloning and PCR detecting YVN | 522 bp |
|  | /cggtcggcatctactctgcagCTACTCGATGTTGTGGCGGAT |  |  |
| *BbGap1* + YVC_ P1_F/R | atcATGCTTCTCTTACCCCCATACTTG | Cloning and PCR detecting *BbGap1* | 1293 bp |
|  | /ACGGGTACCTTGCACCATAACATTACAAGCAGC |  |  |
| *BbGap1* + YVC_ P2_F/R | gtaatgttatggtgcaaGGTACCCGTCCGGCGTGCA | Cloning and PCR detecting YVC | 255 bp |
|  | /cggtcggcatctactactagtCTTGTACAGCTCGTCCATGCC |  |  |
| *BbGap1*+GFP_P1_F/R | gaacctttaatcATGCTTCTCTTACCCCCATACTTG/ tcaccatGCCGCCTCCGCCGCCTCCGCCGCCTTGCACCATAACATTACAAGCAGCGA | Cloning and PCR detecting *BbGap1* | 1293 bp |
| *BbGap1*+GFP_P2_F/R | ATGGTGAGCAAGGGCGAGGAG/ cggtcggcatctactTTACTTGTACAGCTCGTCCATGCC | Cloning and PCR detecting GFP | 717 bp |
| 3HA*+BbGap1*+ _P1_F/R | AcaaccttcaaagagctcATGTACCCATACGATGTTC/ GTAAGAGAAGCATagcgtaatctggaacgtc | Cloning and PCR detecting 3HA | 90 bp |
| 3HA*+BbGap1*+ _P2_F/R | ATGCTTCTCTTACCCCCAT/ tttgtctcaaccccgggTTGCACCATAACATTA | Cloning and PCR detecting *BbGap1* | 1293 bp |
| *BbSdu1* + YVC_ P1_F/R | acctttaatcATGCAATCACCACCTTATCGTTC/  gacgggtaccCGAACGGGAACCGGGCGT | Cloning and PCR detecting BbSdu1 | 852 bp |
| *BbSdu1* + YVC_ P2_F/R | ttcccgttcgGGTACCCGTCCGGCGTGC/  cggtcggcatctactactagtCTTGTACAGCTCGTCCATGCC | Cloning and PCR detecting YVC | 255 bp |
| ORF5+FLAG_P1_F/R | CaagaacctttaatcgaattcATGCCTTTTCTTGGCACCCA/ CTGGCCAAAGACAGGGCC | Cloning and PCR detecting ORF5 | 510 bp |
| ORF5+FLAG_P2_F/R | caagaacctttaatcgaattcATGCCTTTTCTTGGCACCCA-CATGGACTACAAAGACCATG/ cggtcggcatctactctgcagCTTGTCATCGTCATCCTTGTAGTCG | Cloning and PCR detecting 3FLAG | 66 bp |
| *BbSdu1*+GFP_P1_F/R | aacacaaccttcaaagagctcATGCAATCACCACCTTATCGTTC/ gcccttgctcaccatgagctcCGAACGGGAACCGGGCGT | Cloning and PCR detecting BbSdu1 | 942 bp |
| *BbSdu1*+GFP_P2_F/R | ccggttcccgttcggagctcATGGTGAGCAAG/ catttgtctcaaccccgggTCACTTGTACAGCTCGTCCATg | Cloning and PCR detecting GFP | 717 bp |
