# Supplementary material for: A mycovirus enhances fitness of an insect pathogenic fungus and potentially modulates virulence through interactions between viral and host proteins
Source: PLoS Pathog. 2025 Oct 23;21(10):e1013634. doi: 10.1371/journal.ppat.1013634 (PMC12574890; doi:10.1371/journal.ppat.1013634)
Supplement: S5 Table — (DOCX) [file ppat.1013634.s016.docx]

**S5 Table.** Paired primers used for PCR, RT-PCR, and RT-qPCR of ORF1-8.

| Primers | Paired sequences (5'-3') | Purpose | Products |
| --- | --- | --- | --- |
| ex-ORF1_F/R | caagaacctttaatcgaattcATGGCTGCTACCTCAGTGTCATC | Cloning and PCR detecting ORF1 | 2307 bp |
|  | /cggtcggcatctactctgcagTTAGTCGATCATGACTCCGGC |  |  |
| ex-ORF2_F/R | caagaacctttaatcgaattcATGGCAGACCTAACACGTCTGC | Cloning and PCR detecting ORF2 | 2100 bp |
|  | /cggtcggcatctactctgcagTTAACTTGGTATGCTGGCCGC |  |  |
| ex-ORF3_F/R | caagaacctttaatcgaattcATGTTTCGAAGGGAAAGGAACG | Cloning and PCR detecting ORF3 | 1854 bp |
|  | /cggtcggcatctactctgcagTCAGCGACACATCATCCCCA |  |  |
| ex-ORF4_F/R | caagaacctttaatcgaattcATGTCGCTCCACGATGTCATT | Cloning and PCR detecting ORF4 | 798 bp |
|  | /cggtcggcatctactctgcagCTATTTGCCCGCGGCCTCGGT |  |  |
| ex-ORF5_F/R | caagaacctttaatcgaattcATGCCTTTTCTTGGCACCCA | Cloning and PCR detecting ORF5 | 513 bp |
|  | /cggtcggcatctactctgcagTTACTGGCCAAAGACAGGGC |  |  |
| ex-ORF6_F/R | caagaacctttaatcgaattcATGTCTGAAGTATCCTCTTTCGTCC | Cloning and PCR detecting ORF6 | 609 bp |
|  | /cggtcggcatctactctgcagCTATTTCTTGCTTCGTAGTACATG |  |  |
| ex-ORF7_F/R | caagaacctttaatcgaattcATGCCTTTTCTTGGCACCCA | Cloning and PCR detecting ORF7 | 462 bp |
|  | /cggtcggcatctactctgcagCTAACTGGGGAATCCGGGCA |  |  |
| ex-ORF8_F/R | caagaacctttaatcgaattcATGCCTTTTCTTGGCACCCAC | Cloning and PCR detecting ORF8 | 345 bp |
|  | /cggtcggcatctactctgcagTTACTGGCCAAAGACAGGGC |  |  |
| ex-ORF1_RNA_F/R | CAGGCGGATGCTGTGTTTATTG | RT-PCR detecting ORF1 | 599 bp |
|  | /TGGTCTTTCCTATCGGTGAC |  |  |
| exORF2_RNA_F/R | GATGGTGAATGGGATCGCGAGA | RT-PCR detecting ORF2 | 485 bp |
|  | /GAGTCATAGGAGTACCGTGTGA |  |  |
| ex- ORF3_RNA_F/R | ACACTGTTTGAGTACGGCTTCG | RT-PCR detecting ORF3 | 389 bp |
|  | /TCCTCATTGTCCTGGAGATACT |  |  |
| ex- ORF4_RNA_F/R | ATCACGATCAACGCGTGGTCCT | RT-PCR detecting ORF4 | 410 bp |
|  | /TCAATGGCTCCACGCTTGTTCG |  |  |
| ex- ORF5_RNA_F/R | CAATCCAGCGTTTTCGTTGGTG | RT-PCR detecting ORF5 | 349 bp |
|  | /CAGACACACGGTCAATGAACAT |  |  |
| ex- ORF6_RNA_F/R | GTCATTTACCCACCAACCAGGC | RT-PCR detecting ORF6 | 345 bp |
|  | /TACGGCCTCATCCACCGTAGGTA |  |  |
| ex- ORF7_RNA_F/R | CAATCCAGCGTTTTCGTTGGTG | RT-PCR detecting ORF7 | 334 bp |
|  | /CAACGACGGACCACCCTCGT |  |  |
| ex- ORF8_RNA_F/R | ATGCCTTTTCTTGGCACCCAC | RT-PCR detecting ORF8 | 305 bp |
|  | /GACAACACCGCGACGAGCA |  |  |
