# Supplementary material for: A mycovirus enhances fitness of an insect pathogenic fungus and potentially modulates virulence through interactions between viral and host proteins
Source: PLoS Pathog. 2025 Oct 23;21(10):e1013634. doi: 10.1371/journal.ppat.1013634 (PMC12574890; doi:10.1371/journal.ppat.1013634)
Supplement: S6 Table — (DOCX) [file ppat.1013634.s017.docx]

**S6 Table.** Paired primers used for PCR of ORF1-8 + FLAG.

| Primers | Paired sequences (5'-3') | Purpose | Products |
| --- | --- | --- | --- |
| ORF1+FLAG_P1_F/R | caagaacctttaatcgaattcATGGCTGCTACCTCAGTGTCATC  /GTCGATCATGACTCCGGCTTGT | Western blot detecting ORF1 | 86.44 kDa |
| ORF1+FLAG_P2_F/R | aagccggagtcatgatcgacGGCGGCGGAGGCGGCGGAGGCGGC |  |  |
|  | -ATGGACTACAAAGACCATG |  |  |
|  | /cggtcggcatctactctgcagCTTGTCATCGTCATCCTTGTAGTCG |  |  |
| ORF2+3FLAG_P1_F/R | caagaacctttaatcgaattcATGGCAGACCTAACACGTCTGC | Western blot detecting ORF2 | 78.01 kDa |
|  | /ACTTGGTATGCTGGCCGCAACA |  |  |
| ORF2+FLAG_P2_F/R | ttgcggccagcataccaagtGGCGGCGGAGGCGGCGG  -AGGCGGCATGGACTACAAAGACCATG |  |  |
|  | /ACTTGGTATGCTGGCCGCAACA |  |  |
| ORF3+FLAG_P1_F/R | tggtggggatgatgtgtcgcGGCGGCGGAGGCGGCGGAGGC | Western blot detecting ORF3 | 69.73 kDa |
|  | /GCGACACATCATCCCCACCATTTCCT |  |  |
| ORF3+3FLAG_P2_F/R | tggtggggatgatgtgtcgcGGCGGCGGAGGCGGCGGAGGC |  |  |
|  | -GGCATGGACTACAAAGACCATG |  |  |
|  | /cggtcggcatctactctgcagCTTGTCATCGTCATCCTTGTAGTCG |  |  |
| ORF4+FLAG_P1_F/R | caagaacctttaatcgaattcATGTCGCTCCACGATGTCATT | Western blot detecting ORF4 | 31.87 kDa |
|  | /GCCTTTGCCCGCGGCCTCGGTGGCGGC |  |  |
| ORF4+FLAG_P2_F/R | ccaccgaggccgcgggcaaaggcGGCGGAGGCGGCGGAGGCGGC |  |  |
|  | -ATGGACTACAAAGACCATG |  |  |
|  | /cggtcggcatctactctgcagCTTGTCATCGTCATCCTTGTAGTCG |  |  |
| ORF5+3FLAG_P1_F/R | caagaacctttaatcgaattcATGCCTTTTCTTGGCACCCA | Western blot detecting ORF5 | 22.1 kDa |
|  | /CTGGCCAAAGACAGGGCC |  |  |
| ORF5+FLAG_P2_F/R | caagaacctttaatcgaattcATGCCTTTTCTTGGCACCCA |  |  |
|  | -CATGGACTACAAAGACCATG |  |  |
|  | /cggtcggcatctactctgcagCTTGTCATCGTCATCCTTGTAGTCG |  |  |
| ORF6+FLAG_P1_F/R | caagaacctttaatcgaattcATGTCTGAAGTATCCTCTTTCGTCC | Western blot detecting ORF6 | 25.47 kDa |
|  | / GCCTTTCTTGCTTCGTAGTACATG |  |  |
| ORF6+FLAG_P2_F/R | atgtactacgaagcaagaaaggcGGCGGAGGCGGCGGAGGCGGCA |  |  |
|  | -TGGACTACAAAGACCATG |  |  |
|  | /cggtcggcatctactctgcagCTTGTCATCGTCATCCTTGTAGTCG |  |  |
| ORF7+FLAG_P1_F/R | caagaacctttaatcgaattcATGCCTTTTCTTGGCACCCA | Western blot detecting ORF7 | 20.67 kDa |
|  | /ACTGGGGAATCCGGGCAGGCAG |  |  |
| ORF7+FLAG_P2_F/R | gcctgcccggattccccagtGGCGGCGGAGGCGGCGGAGGCGGCA |  |  |
|  | -TGGACTACAAAGACCATG |  |  |
|  |  |  |  |
|  | |  |  |
| **S6 table.** (continued) | |  |  |
| Primers | Paired sequences (5'-3') | Purpose | Products |
|  | /cggtcggcatctactctgcagCTTGTCATCGTCATCCTTGTAGTCG |  |  |
| ORF8+3FLAG_P1_F/R | caagaacctttaatcgaattcATGCCTTTTCTTGGCACCCA | Western blot detecting ORF8 | 15.44 kDa |
|  | /CTGGCCAAAGACAGGGCCCTGAAC |  |  |
| ORF8+3FLAG_P2_F/R | agggccctgtctttggccagGGCGGCGGAGGCGGCGGAGGCGGC |  |  |
|  | -ATGGACTACAAAGACCATG |  |  |
|  | /cggtcggcatctactctgcagCTTGTCATCGTCATCCTTGTAGTCG |  |  |
