# Supplementary material for: A mycovirus enhances fitness of an insect pathogenic fungus and potentially modulates virulence through interactions between viral and host proteins
Source: PLoS Pathog. 2025 Oct 23;21(10):e1013634. doi: 10.1371/journal.ppat.1013634 (PMC12574890; doi:10.1371/journal.ppat.1013634)
Supplement: S7 Table — (DOCX) [file ppat.1013634.s018.docx]

**S7 Table.** Paired primers used for manipulation of target genes in Bb2151.

| Primers | Paired sequences (5'-3') | Purpose | Products |
| --- | --- | --- | --- |
| up*BbGap1*-F/R | ttcctgcagcccgggggatccCGATACTCTGAGAAAGAATCTCGGC | Cloning 5' *BbGap1* for | 614 bp |
|  | /tcatcttctgtcgacggatccCTTATGTCGATTGCGATTCAGG | targeted gene disruption |  |
| dn*BbGap1*-F/R | atctgatgaactagttctagaTGATATACCAAGACGACACGTAAAGTA | Cloning 3' *BbGap1* for | 845 bp |
|  | /cgcggtggcggccgctctagaGACCATTTCCATTCTCCAACTATCA | targeted gene disruption |  |
| *BbGap1-gfp*-F/R | cttcccatccaagaacctttaatcATGCTTCTCTTACCCCCATACTTG | Cloning *BbGap1* cDNA | 1296 bp |
|  | /tcaccatgccgcctccgccgcctccgccgccTTGCACCATAACATTACAAGCAGCGA | for fusion to *gfp* |  |
| M-*BbGap1*-F/R | AGTCTGTTCTCCGATACTCGTC/TGGGCGTGGGCGTGGGCTT | PCR detecting *BbGap1* | 448 bp |
| *Bar-* F/R | TCGTCAACCACTACATCGAGAC/GAAGTCCAGCTGCCAGAAAC | PCR detecting *Bar* | 434 bp |
| up*BbSdu1*-F/R | cgaattcctgcagcccgggggatccATCCACTGCTTGAAGT/ tcatcttctgtcgacggatccCGTTGATTTCTGACAATCGGTCT | Cloning and PCR detecting BbSdu1 upstream | 1051 bp |
| down*BbSdu1*-F/R | atctgatgaactagttctagaGCATGGTAGTAATGGGCATGG/ cgcggtggcggccgctctagaGCGGTATCCCTTACAGTAGTGCC | Cloning and PCR detecting BbSdu1 dowmstream | 893 bp |
| *BbSdu1* F/R | TGTAACTTACAGCCACTGTGGC/TATGCGGTGAAATGATTGCAGT | PCR detecting BbSdu1 | 680 bp |
| Complementation | cttgatatcgaattcctgcagATTGGCCATCATCTTCTTATCGTA/ aggccccctgtcgagctgcagTCATATGGCTAGATCATAAATCCATGC | Cloning and PCR detecting native Promoter and *BbSdu1* full length | 2387 bp |
| ORF5-RNAi-F/R | GGATTGACAACCGCGGATC | PCR detecting ORF5 partial  sequence | 352 bp |
|  | GGCACCCACCTGCCCCCT |  |  |
